# Plastome structure, phylogenomic analyses and molecular dating of Arecaceae

**DOI:** 10.3389/fpls.2022.960588

**Published:** 2022-09-27

**Authors:** Da-Juan Chen, Jacob B. Landis, Hong-Xin Wang, Qing-Hui Sun, Qiao Wang, Hua-Feng Wang

**Affiliations:** ^1^ Hainan Yazhou Bay Seed Laboratory, Sanya Nanfan Research Institute of Hainan University, Sanya, China; ^2^ Collaborative Innovation Center of Nanfan and High-Efficiency Tropical Agriculture, Hainan University, Haikou, China; ^3^ School of Integrative Plant Science, Section of Plant Biology and the L.H. Bailey Hortorium, Cornell University, Ithaca, NY, United States; ^4^ BTI Computational Biology Center, Boyce Thompson Institute, Ithaca, NY, United States; ^5^ Zhai Mingguo Academician Work Station, Sanya University, Sanya, China; ^6^ Hainan Shengda Modern Agriculture Development Co., Ltd., Qionghai, China

**Keywords:** Arecaceae, chloroplast genome, phylogeny, adaptive evolution, molecular dating

## Abstract

Arecaceae is a species-rich clade of Arecales, while also being regarded as a morphologically diverse angiosperm family with numerous species having significant economic, medicinal, and ornamental value. Although in-depth studies focused on the chloroplast structure of Arecaceae, as well as inferring phylogenetic relationships using gene fragments, have been reported in recent years, a comprehensive analysis of the chloroplast structure of Arecaceae is still needed. Here we perform a comprehensive analysis of the structural features of the chloroplast genome of Arecaceae, compare the variability of gene sequences, infer phylogenetic relationships, estimate species divergence times, and reconstruct ancestral morphological traits. In this study, 74 chloroplast genomes of Arecaceae were obtained, covering five subfamilies. The results show that all chloroplast genomes possess a typical tetrad structure ranging in size between 153,806-160,122 bp, with a total of 130-137 genes, including 76-82 protein-coding genes, 29-32 tRNA genes, and 4 rRNA genes. Additionally, the total GC content was between 36.9-37.7%. Analysis of the SC/IR boundary indicated that the IR region underwent expansion or contraction. Phylogenetic relationships indicate that all five subfamilies in Arecaceae are monophyletic and that Ceroxyloideae and Arecoideae are sister groups (BS/PP = 100/1). The results of molecular dating indicate that the age of the crown group of Arecaceae is likely to be 96.60 [84.90-107.60] Ma, while the age of the stem group is 102.40 [93.44-111.17] Ma. Reconstruction of ancestral traits indicate that the ancestral characteristics of the family include monoecious plants, one seed, six stamens, and a smooth pericarp.

## Introduction

Arecaceae is a family belonging to the Arecales, ranking fifth among monocot families in terms of species richness. According to APG IV (Angiosperm Phylogeny Group et al., 2016), Arecaceae is divided into five subfamilies (Calamoideae, Nypoideae, Coryphoideae, Ceroxyloideae, and Arecoideae), with 181 genera and approximately 2600 species currently recognized (Dransfield et al., 2005; Dransfield et al., 2008; Baker and Dransfield, 2016). Members of Arecaceae are widely distributed in tropical and subtropical regions all over the world (Govaerts and Dransfield, 2005; Dransfield et al., 2008; Baker et al., 2009; Trias-Blasi et al., 2015), with only a few species extending into temperate regions. Arecoideae, the largest and most diverse subfamily in Arecaceae, includes approximately 60% of the genera and 50% of the species in Arecaceae (Dransfield et al., 2008). The family originated in North America, with subsequent diversification of most tribes having occurred in the Americas (Comer et al., 2016).

**Figure 1:**
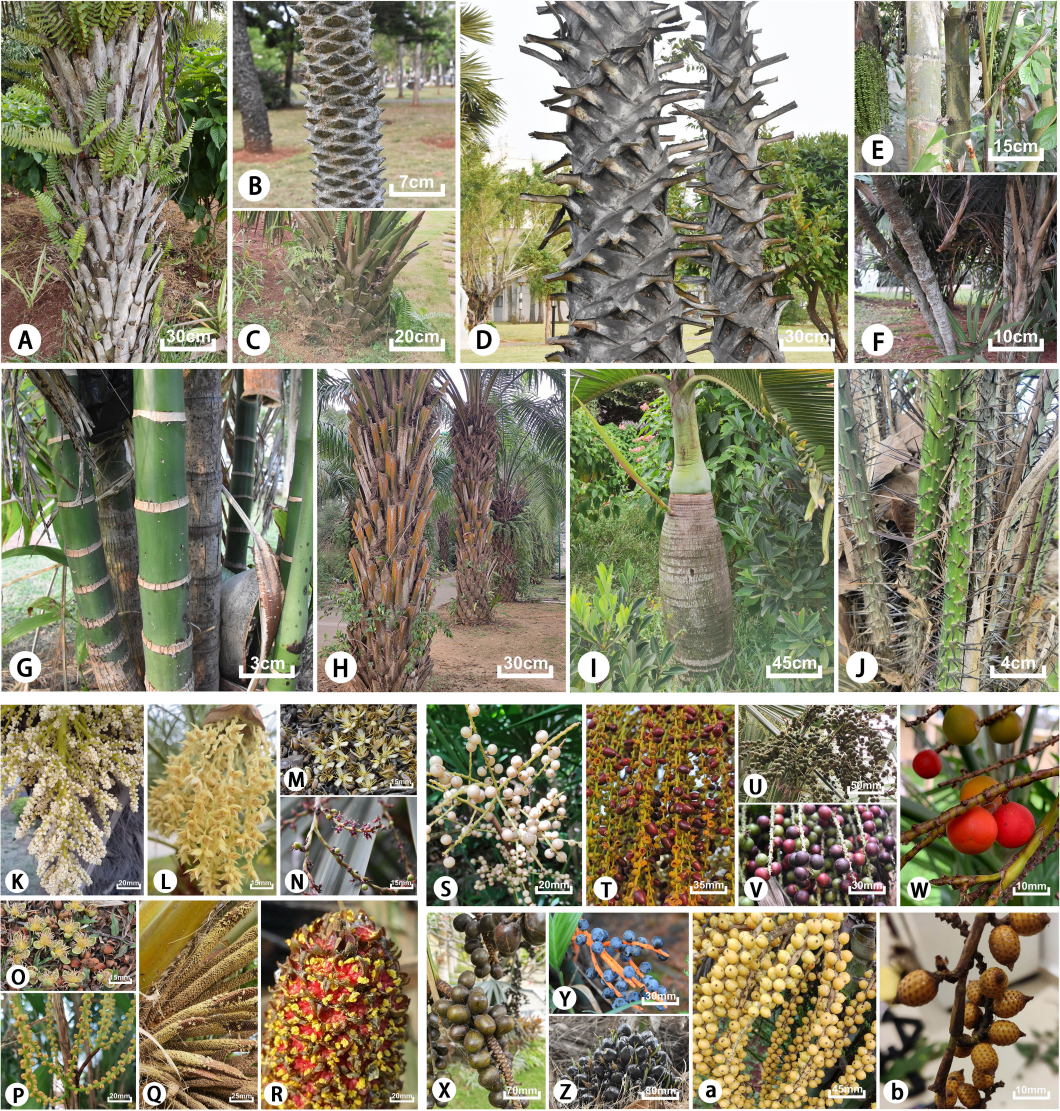
Typical morphological characteristics of tribes of the subfamilies in Arecacea. Coryphoideae: **(A)** *Copernicia alba*; **(B, L, T)** *Phoenix roebelenii*; **(C, U)** *Sabal minor*; **(D)** *Borassus flabellifer*; **(E, M, V)** *Caryota mitis*; **(F, N, W)** *Chuniophoenix hainanensis*; **(K)** *Trachycarpus fortunei*; **(S)** *Lanonia dasyantha*; **(X)** *Bismarckia nobilis*; Arecoideae: **(G)** *Areca triandra*; **(H, Q, Z)** *Elaeis guineensis*; **(I)** *Hyophorbe lagenicaulis*; **(O)** *Wodyetia bifurcata*; **(P, Y)** *Chamaedorea elegans*; **(a)** *Ptychosperma macarthurii*; Calamoideae: **(J, R)** *Salacca zalacca*; **(b)** *Calamus rhabdocladus*.

Arecaceae is also one of the most morphologically diverse angiosperm groups with a variety of morphological characteristics (Dransfield et al., 2008). Most typical forms are non-branching arbors, some are shrubs, and very few species are lianas or without aboveground stems. The surface of the plant stem is smooth, rough, or spiny, and covered with remnants of old petiole bases or leaf scars; the inflorescence is usually large and multi-branched or surrounded by spathes; the shape and size of the fruits are diverse (**Figure 1**). Thomas and De Franceschi (2013) noted that each of the five subfamilies and their tribes have distinct distinguishing features. Arecaceae is a large economic family comparable to Gramineae, including food crops, oil crops, sugar crops, fruits, and other economic crops with great value, such as *Cocos nucifera*, *Phoenix dactylifera*, and *Elaeis guineensis* (Dransfield et al., 2008; Fadini et al., 2009). Numerous species have been widely cultivated as ornamentals and are indispensable species to courtyards and road landscapes (Wang et al., 2014; Tang et al., 2018). There are also some edible and medicinal plants, such as *Cocos nucifera* in Arecoideae, which have important medicinal value such as a therapeutic effect on diabetes (Joseph et al., 2019), while *Butia eriospatha* is not only used for ornamental purposes but also has edible fruits (de Souza Magnabosco et al., 2020). Plants of Arecaceae also have great anthropogenic uses, such as leaves, fruits, seeds, and fibers serving as a basic public resource for traditional communities and which have even been exploited commercially on a large scale (Johnson, 2010; Kissling et al., 2019).

Members of Arecaceae have nearly every possible combination of hermaphrodite or unisexual flowers observed among the numerous angiosperm families, and it is this feature that makes the family a great system for studying the evolution of plant sexuality. However, in most evolutionary studies, the sampling has been relatively small with only a few chloroplast fragments used. Therefore, the phylogenetic relationship of Arecaceae at the subfamily level are still not clear, with support of some nodes being quite low and the phylogenetic position of some taxa remaining controversial. Wang (2007) inferred the phylogenetic relationships among 18 genera of native Chinese Arecaceae using sequences of the *matK* intron and ribosomal transcribed spacer (ITS). The results showed that the ITS sequences of some Arecaceae plants were drastically different within species, with some conspecific plants being more different than those of non-conspecific plants. Asmussen et al. (2000) constructed a phylogenetic relationship for 65 Arecaceae species by combining the *rps16* intron and *trnL-trnF* region and found that the Nypoideae + Calamoideae clade formed a sister group to the rest of the family with moderate support. Comer et al. (2015) studied the phylogenetic relationships of Arecoideae using plastids obtained by next-generation sequencing and showed that Arecoideae was monophyletic with the tribe Chamaedoreaeae diverging first. That study identified three major evolutionary clades in the group: the Reinhardtieae, Roystoneeae, Cocoseae (RRC) clade, the Podococceae, Oranieae, Sclerospermeae (POS) clade, and the core arecoids clade (Areceae, Euterpeae, Geonomateae, Leopoldinieae, Manicarieae, Pelagodoxeae). Baker et al. (2009; 2011) also supported Arecoideae as monophyletic, but the tribe Iriarteeae was found to be the earliest divergent lineage. The nuclear genome phylogeny inferred by Comer et al. (2016) also supports the tribe Iriarteeae as the earliest divergent lineage. However, in the study by Pichardo-Marcano et al. (2019) using three nuclear and one chloroplast DNA markers to infer a Bayesian maximum clade credibility tree, the results showed that the tribes Chamaedoreaeae and Iriarteeae are sister groups to each other. Bacon et al. (2016) generated data from five plastid (*ndhA*, *petD-rpoA*, *psbK-trnS*, *trnG* and *trnD-trnT*) and six nuclear (*AG1*, *CISPs 4* and *5*, *PRK*, *RPB2* and *WRKY21*) loci to infer the phylogenetic relationships of tribe Iriarteeae using Bayesian analysis, showing that all genera were inferred to be monophyletic and the affinities between genera were strongly to moderately supported. Based on the four plastid intergenic spacers (*psbA-trnH, psbZ-trnfM, atpI-atpH, and rps3-rpl16*), Faye et al. (2014) performed a phylogenetic analysis on the species-level subtribe Ancistrophyllinae in the Calamoideae using maximum parsimony, maximum likelihood and Bayesian analysis. The results showed that the Ancistrophyllinae and genera within the subtribe are strongly supported as monophyletic. Barrett et al. (2019) analyzed the phylogenetic relationships, biogeography and evolution of *Brahea* in the Coryphoideae, the ML and Bayesian analysis of whole aligned plastomes strongly supported *Brahea* as monophyletic. The majority of previous studies are based on the analysis of plastids at the tribe/genera level, while rarely analyzing the phylogenetic relationship at the subfamily level (Hahn, 2002b; Faye et al., 2014; Heyduk et al., 2016; Barrett et al., 2019).

Although some plastomes of Arecaceae have been reported, most studies have focused on the genus or species level, and no comprehensive plastome analysis of Arecaceae has been carried out. Therefore, based on extensive sampling around the world, this study uses chloroplast genome data to address the following three scientific goals: 1. Analyze the chloroplast genome structure of Arecaceae; 2. Establish robust phylogenetic relationships of Arecaceae at the subfamily level; 3. Estimate the divergence time of each subfamily through and combine with existing traits to reconstruct ancestral traits of Arecaceae species.

## Materials and methods

### Taxon sampling, DNA extraction and sequencing

In this study, leaf material of 24 species of Arecaceae, covering three subfamilies and 22 genera, were collected, and the leaves were stored in silica gel. Total genomic DNA was extracted from silica-dried leaf material using a modified cetyltrimethyl ammonium bromide (CTAB) method (Doyle and Doyle, 1987). Quality and quantity of the DNA was assessed using 1% agarose gel electrophoresis and an ultra-micro spectrophotometer (ultra-micro nucleic acid analyzer). Before library construction and whole genome sequencing of DNA, we quantified and analyzed each sample using an Agilent 2100 BioAnalyzer (Davis, California, USA), and selected DNA samples with a total content of at least ≥0.8 ug. We constructed paired-end sequencing libraries with an insert size of 300-500 bp and performed sequencing using the BGISEQ-500 platform at the Beijing Genome Research Institute (BGI; Shenzhen, China). Raw reads were filtered and trimmed using SOAPfilter_v2.2 with the following standard parameters: (1) screening for low quality base reads (>10% Ns and/or >40% low quality bases); (2) screening for reads generated by PCR duplication; (3) trimming of adapter sequences. All newly sequenced raw reads have been submitted to the Sequence Read Archive (SRA) under BioProject PRJNA748537 (see **Table 1** for details such as collection location, GenBank number, etc.). The collection of the 24 newly sequenced samples was approved by Hainan University (Hainan, China) and complied with local policy requirements. In addition, we downloaded 50 species of Arecaceae (covering five subfamilies) and three species of Asparagaceae as outgroups from the National Center for Biotechnology Information (NCBI), the details are shown in **Table 2**. Thus, a total of 74 Arecaceae chloroplast genomes representing five subfamilies and 54 genera were used for analyses.

**Table 1:** GenBank number, SRA number and collection location information of 24 newly sequenced chloroplast genomes in Arecaceae.

Species name	Sub family	Accession number	SRA accessions	Specimen collection number	Locality	Latitude and Longitude
Areca triandra	Arecoideae	OL674129	SRR18094486	HUTB, P10	China, Hainan, Haikou	19°50'43"N,110°26'58"E
Dictyosperma album	Arecoideae	OL674132	SRR18094474	HUTB, P15	China, Hainan, Haikou	19°50'43"N,110°26'58"E
Dypsis madagascariensis	Arecoideae	OL674131	SRR18094464	HUTB, P14	China, Hainan, Haikou	19°50'43"N,110°26'58"E
Hyophorbe lagenicauli	Arecoideae	OL674134	SRR18094461	HUTB, P17	China, Hainan, Haikou	19°50'43"N,110°26'58"E
Hyophorbe verschaffeltii	Arecoideae	OL674135	SRR18094460	HUTB, P18	China, Hainan, Haikou	19°50'43"N,110°26'58"E
Pinanga coronata	Arecoideae	OL674142	SRR18094459	HUTB, P11	China, Hainan, Haikou	19°50'43"N,110°26'58"E
Ptychosperma macarthurii	Arecoideae	OL674128	SRR18094458	HUTB, P9	China, Hainan, Haikou	19°50'43"N,110°26'58"E
Veitchia merrillii	Arecoideae	OL674130	SRR18094484	HUTB, P13	China, Hainan, Haikou	19°50'43"N,110°26'58"E
Verschaffeltia splendida	Arecoideae	OL674140	SRR18094483	HUTB, P12	China, Hainan, Haikou	19°50'43"N,110°26'58"E
Wodyetia bifurcata	Arecoideae	OL674133	SRR18094482	HUTB, P16	China, Hainan, Haikou	19°50'43"N,110°26'58"E
Euterpe oleracea	Arecoideae	OL674119	SRR18094462	HUTB, A29	China, Hainan, Qionghai	19°24'5"N,110°28'46"E
Raphia vinifera	Calamoideae	OL674136	SRR18094481	HUTB, P19	China, Hainan, Haikou	19°50'43"N,110°26'58"E
Salacca zalacca	Calamoideae	OL674120	SRR18094480	HUTB, A91	China, Hainan, Qionghai	19°24'5"N,110°28'46"E
Calamus faberi	Calamoideae	OL674137	SRR18094469	HUTB, W17	China, Hainan, Wuzhishan	18°46′38″N,109°38′38″E
Calamus jenkinsianus	Calamoideae	OL674138	SRR18094468	HUTB, W19	China, Hainan, Wuzhishan	18°50′41″N,109°40′43″E
Acoelorraphe wrightii	Coryphoideae	OL674123	SRR18094479	HUTB, P3	China, Hainan, Haikou	19°50'43"N,110°26'58"E
Bismarckia nobilis	Coryphoideae	OL674126	SRR18094478	HUTB, P7	China, Hainan, Haikou	19°50'43"N,110°26'58"E
Chuniophoenix hainanensis	Coryphoideae	OL674121	SRR18094477	HUTB, A243	China, Hainan, Ledong	18°43'50"N,108°54'33"E
Copernicia alba	Coryphoideae	OL674124	SRR18094476	HUTB, P4	China, Hainan, Haikou	19°50'43"N,110°26'58"E
Latania lontaroides	Coryphoideae	OL674141	SRR18094475	HUTB, A317	China, Hainan, Ledong	18°43'50"N,108°54'33"E
Latania verschaffeltii	Coryphoideae	OL674125	SRR18094473	HUTB, P6	China, Hainan, Haikou	19°50'43"N,110°26'58"E
Phoenix roebelenii	Coryphoideae	OL674127	SRR18094472	HUTB, P8	China, Hainan, Haikou	19°50'43"N,110°26'58"E
Pritchardia pacifica	Coryphoideae	OL674139	SRR18094471	HUTB, A252	China, Hainan, Ledong	18°43'50"N,108°54'33"E
Sabal minor	Coryphoideae	OL674122	SRR18094470	HUTB, P2	China, Hainan, Haikou	19°50'43"N,110°26'58"E

**Table 2:** Summary of the main characteristics of plastomes in Arecaceae and related outgroups.

Species name	Subfamily	Accession number	Genome size and GC content								Number of genes			
			Total		LSC		SSC		IR					
			Length (bp)	G+C (%)	Length (bp)	G+C(%)	Length (bp)	G+C(%)	Length (bp)	G+C(%)	Gene	CDS	tRNA	rRNA
Archontophoenix alexandrae	Arecoideae	NC_046017.1	159196	37.20	87055	35.30	17763	30.80	27189	42.40	132	80	30	4
Areca catechu	Arecoideae	NC_050163.1	158689	37.30	86814	35.30	17601	31.10	27137	42.50	131	80	29	4
Areca triandra	Arecoideae	P10	158339	37.50	86633	35.50	17494	31.30	27106	42.60	131	80	29	4
Dictyosperma album	Arecoideae	P15	157892	37.30	86890	35.30	17584	31.00	26709	42.70	130	80	29	4
Dypsis madagascariensis	Arecoideae	P14	158897	37.30	87159	35.30	17502	31.30	27118	42.50	131	80	29	4
Verschaffeltia splendida H	Arecoideae	P12	158678	37.30	87190	35.30	17378	31.20	27055	42.50	133	80	30	4
Veitchia merrillii	Arecoideae	P13	158692	37.30	86783	35.40	17561	30.90	27174	42.40	133	80	30	4
Pinanga coronata	Arecoideae	P11	157724	37.30	86832	35.30	18380	31.40	26256	42.80	131	80	29	4
Ptychosperma macarthurii	Arecoideae	P9	158079	37.30	86679	35.30	17690	30.80	26855	42.50	132	80	30	4
Wodyetia bifurcata	Arecoideae	P16	159020	37.20	87036	35.30	17652	30.80	27166	42.40	133	80	30	4
Hyophorbe lagenicaulis	Arecoideae	P17	157676	37.20	85841	35.20	17597	30.40	27119	42.50	132	80	30	4
Hyophorbe verschaffeltii	Arecoideae	P18	157712	37.20	85937	35.20	17513	30.50	27131	42.50	132	80	30	4
Chamaedorea elegans	Arecoideae	NC_051509.1	156922	37.30	85413	35.30	17209	30.60	27150	42.60	132	80	30	4
Acrocomia aculeata	Arecoideae	NC_037084.1	155829	37.50	84265	35.70	17380	31.20	27092	42.50	133	80	30	4
Astrocaryum aculeatum	Arecoideae	NC_044482.1	156804	37.40	85037	35.50	17605	31.00	27081	42.50	133	81	30	4
Astrocaryum murumuru	Arecoideae	NC_044481.1	156801	37.40	85017	35.50	17622	31.00	27081	42.50	133	81	30	4
Cocos nucifera	Arecoideae	NC_022417.1	154731	37.40	84092	35.50	17391	31.10	26555	42.60	133	80	30	4
Elaeis guineensis	Arecoideae	NC_017602.1	156973	37.40	85192	35.50	17639	31.00	27071	42.50	137	80	30	4
Syagrus coronata	Arecoideae	NC_029241.1	155053	37.50	84409	35.50	17474	31.10	26522	42.60	135	79	32	4
Butia eriospatha	Arecoideae	NC_058633.1	154048	37.50	83805	35.60	17369	31.20	26437	42.70	137	82	30	4
Bactris gasipaes var. chichagui	Arecoideae	NC_058634.1	156646	37.50	85118	35.50	17452	31.30	27038	42.60	132	80	30	4
Euterpe edulis	Arecoideae	NC_057602.1	158397	37.40	86716	35.40	17403	31.40	27139	42.50	133	80	30	4
Euterpe oleracea	Arecoideae	A29	159237	37.30	87250	35.30	17755	30.90	27116	42.50	133	80	30	4
Podococcus barteri	Arecoideae	NC_027276.1	157688	37.70	85472	35.80	17721	31.60	27220	42.60	136	82	30	4
Calamus faberi	Calamoideae	W17	157106	37.40	85497	35.40	17199	31.40	27205	42.40	133	80	30	4
Calamus jenkinsianus	Calamoideae	W19	158029	37.30	85923	35.30	17522	31.30	27292	42.30	133	80	30	4
Calamus caryotoides	Calamoideae	NC_020365.1	157270	37.40	85525	35.30	17595	31.20	27075	42.40	133	80	30	4
Metroxylon warburgii	Calamoideae	NC_029959.1	157516	37.40	85400	35.40	17553	31.30	27281	42.30	133	80	30	4
Pigafetta elata	Calamoideae	NC_029956.1	157708	37.40	85599	35.40	17533	31.40	27288	42.30	133	81	30	4
Salacca ramosiana	Calamoideae	NC_029954.1	157047	37.40	85121	35.40	17594	31.30	27166	42.40	133	80	30	4
Salacca zalacca	Calamoideae	A91	157723	37.30	85634	35.30	17723	31.20	27183	42.40	133	80	30	4
Eugeissona tristis	Calamoideae	NC_029963.1	155304	37.70	85080	35.60	13768	31.60	28228	42.30	132	76	30	4
Eremospatha macrocarpa	Calamoideae	NC_029964.1	154031	37.70	83583	35.90	17180	31.50	26634	42.50	133	80	30	4
Mauritia flexuosa	Calamoideae	NC_029947.1	156367	37.50	85984	35.60	17749	31.30	26007	42.70	133	80	30	4
Raphia vinifera	Calamoideae	P19	155203	37.40	84474	35.40	17275	31.30	26727	42.40	133	80	30	4
Phytelephas aequatorialis	Ceroxyloideae	NC_029957.1	159075	37.20	86910	35.30	17639	30.70	27263	42.40	133	80	30	4
Pseudophoenix vinifera	Ceroxyloideae	NC_020364.1	157829	37.30	86046	35.40	17587	30.70	26943	42.60	133	80	30	4
Borassodendron machadonis	Coryphoideae	NC_029969.1	158144	37.40	86137	35.50	17981	31.30	27013	42.50	133	80	30	4
Borassus flabellifer	Coryphoideae	KP901247.1	160021	37.10	87445	35.20	18066	30.50	27255	42.40	133	80	30	4
Bismarckia nobilis	Coryphoideae	P7	159504	37.20	86936	35.30	18110	30.40	27229	42.50	133	80	30	4
Latania verschaffeltii	Coryphoideae	P6	159823	37.10	87476	35.10	17915	30.80	27216	42.50	133	80	30	4
Latania lontaroides	Coryphoideae	A317	160122	37.10	87639	35.00	17901	30.80	27291	42.40	133	80	30	4
Lodoicea maldivica	Coryphoideae	NC_029960.1	159010	37.30	86561	35.40	17877	30.80	27286	42.40	133	80	30	4
Arenga caudata	Coryphoideae	NC_029971.1	159744	36.90	87496	34.80	17776	30.40	27235	42.40	133	80	30	4
Arenga pinnata	Coryphoideae	NC_045907.1	159598	37.00	87142	35.00	17994	30.10	27231	42.40	132	80	30	4
Caryota mitis	Coryphoideae	NC_029948.1	159819	37.10	87534	35.20	17767	30.60	27259	42.40	133	80	30	4
Caryota obtusa	Coryphoideae	NC_054217.1	159882	37.00	87581	34.90	17695	30.60	27271	42.40	130	78	30	4
Caryota urens	Coryphoideae	NC_057595.1	159702	37.00	87502	35.00	17668	30.70	27266	42.40	133	80	30	4
Wallichia densiflora	Coryphoideae	NC_029949.1	159717	36.90	87347	34.90	17920	30.20	27225	42.40	132	79	30	4
Chuniophoenix hainanensis	Coryphoideae	A243	155276	37.20	84951	35.30	17903	30.40	26211	42.50	133	80	30	4
Chuniophoenix nana	Coryphoideae	NC_029966.1	153806	37.50	84283	35.50	17311	31.30	26106	42.70	133	80	30	4
Corypha lecomtei	Coryphoideae	NC_029965.1	154342	37.60	82900	35.70	17191	31.20	27125	42.60	133	80	30	4
Leucothrinax morrisii	Coryphoideae	NC_029961.1	158452	37.30	86572	35.30	17370	31.40	27255	42.40	133	80	30	4
Trithrinax brasiliensis	Coryphoideae	NC_029951.1	158487	37.30	86512	35.30	17457	31.40	27259	42.40	133	81	30	4
Phoenix canariensis	Coryphoideae	NC_051507.1	158477	37.20	86189	35.30	17704	30.80	27292	42.40	133	80	30	4
Phoenix roebelenii	Coryphoideae	P8	158283	37.20	85906	35.30	17743	30.60	27317	42.30	132	80	29	4
Brahea aculeata	Coryphoideae	NC_045079.1	158659	37.20	86532	35.30	17577	31.00	27263	42.40	133	80	30	4
Brahea armata	Coryphoideae	NC_045080.1	158356	37.30	86529	35.30	17535	31.10	27158	42.50	133	80	30	4
Brahea edulis	Coryphoideae	NC_045081.1	158736	37.20	86383	35.30	17838	30.60	27251	42.40	133	80	30	4
Brahea sarukhanii	Coryphoideae	NC_045082.1	158653	37.20	86468	35.30	17779	30.50	27215	42.50	133	80	30	4
Brahea brandegeei	Coryphoideae	NC_029968.1	158733	37.20	86450	35.30	17661	30.60	27311	42.30	133	80	30	4
Colpothrinax cookii	Coryphoideae	NC_028026.1	157867	37.30	85623	35.40	17732	30.70	27256	42.40	133	80	30	4
Copernicia alba	Coryphoideae	P4	157240	37.20	86415	35.10	17503	30.70	26661	42.50	133	80	30	4
Pritchardia pacifica	Coryphoideae	A252	157661	37.30	85794	35.30	17373	31.20	27247	42.40	133	80	30	4
Pritchardia thurstonii	Coryphoideae	NC_029955.1	157909	37.30	85720	35.30	17694	31.00	27247	42.40	133	80	30	4
Acoelorraphe wrightii	Coryphoideae	P3	158509	37.30	86154	35.40	17877	30.70	27239	42.40	133	80	30	4
Serenoa repens	Coryphoideae	NC_029953.1	158952	37.30	86318	35.40	18134	30.60	27190	42.50	133	80	30	4
Chamaerops humilis	Coryphoideae	NC_029967.1	158653	37.20	86233	35.30	17947	30.40	27236	42.40	133	80	30	4
Trachycarpus fortunei	Coryphoideae	NC_053365.1	158613	37.20	86422	35.30	17847	30.60	27172	42.50	131	80	29	4
Trachycarpus nanus	Coryphoideae	NC_057594.1	158713	37.20	86395	35.30	17838	30.60	27240	42.40	133	80	30	4
Washingtonia robusta	Coryphoideae	NC_029974.1	157866	37.40	85641	35.40	17520	31.30	27352	42.30	132	80	29	4
Sabal domingensis	Coryphoideae	NC_026444.1	157835	37.50	85967	35.50	17351	31.60	27258	42.40	132	80	29	4
Sabal minor	Coryphoideae	P2	158848	37.30	86643	35.30	17689	31.00	27258	42.40	132	80	29	4
Nypa fruticans	Nypoideae	NC_029958.1	158391	37.20	86496	35.20	17676	30.70	27179	42.40	133	80	30	4
Ophiopogon bodinieri	Ruscoideae	NC_051508.1	157078	37.60	85374	35.60	18750	31.20	26477	43.00	132	80	30	4
Ophiopogon jaburan	Ruscoideae	NC_049870.1	156454	37.70	85144	35.70	18314	31.70	26498	43.00	132	80	30	4
Dracaena fragrans	Ruscoideae	NC_054234.1	155183	37.50	83703	35.50	18466	31.10	26507	42.90	131	79	30	4

### Plastome assembly and annotation

We used GetOrganelle v1.7.5.0 (Jin et al., 2020) to assemble the chloroplast genomes from the clean reads of each species, with default parameters (see the online manual available at https://github.com/Kinggerm/GetOrganelle). The newly assembled slimmed assembly graph (FASTG) and selected target assembly graph (GFA) were visualized by Bandage v0.8.1 (Wick et al., 2015) to assess the completeness of the final assembly graph. The Mauve v1.1.3 (Darling et al., 2004) alignment was used to check the collinearity of genomic sequences before annotation of the sequences. The plastome sequences were initially annotated with Geneious Prime v2021.2.2 (Kearse et al., 2012), using close relatives as reference sequences, with further manual editing of the start codons, stop codons, and intron/exon boundaries. tRNAscan-SE1.21 was used to verify tRNA genes (Schattner et al., 2005). Plastome maps were drawn with OrganellarGenomeDRAW (OGDRAW) v1.3.1 (see https://chlorobox.mpimp-golm.mpg.de/OGDraw.html) (Lohse et al., 2013). All newly annotated plastomes sequences have been submitted to NCBI (see **Table 1** for GenBank numbers).

### Plastome comparative analysis and sequence differences

Based on the whole chloroplast genome phylogeny in this study (**Figure 5**), we selected 30 representative species (samples were selected by genus, including 21 newly sequenced samples and nine samples downloaded from NCBI, covering five subfamilies) for comparative analysis. Plastome comparisons across the 30 representative species were performed in Shuffle-LAGAN mode on the mVISTA program (see genome.lbl.gov/vista/index.shtml) (Frazer et al., 2004), with *Acrocomia aculeata* (NC_037084.1) as the annotation reference. We used Unipro UGENE v38.1 (Rose et al., 2019) to confirm the IR region. Photoshop was used to draw the IR/SC boundary map of the thirty chloroplast genomes of Arecaceae.

### Phylogenetic analysis

We inferred phylogenetic relationships using 77 species of Arecaceae with three species of Asparagaceae serving as outgroups: *Ophiopogon bodinieri* (NC_051508.1), *Ophiopogon jaburan* (NC_049870.1) and *Dracaena fragrans* (NC_054234.1). Plastome sequences were aligned with MAFFT v7.313 (Katoh and Standley, 2013) and aligned columns with more than 90% missing data were removed using Phyutility (Smith and Dunn, 2008). For ML and BI inference, we generated two datasets, one of the protein-coding sequences (CDSs) and the other with complete plastome sequences. Additionally, we analyzed other data subsets for phylogenetic relationships (i.e. Non-coding regions, LSC, SSC and IRb regions, whole plastome sequence minus one Inverted Repeat copy sequence (No-IRA)). Maximum likelihood analyses were conducted using IQ-TREE v1.6.8 (Nguyen et al., 2015), while searching for the best partition scheme (Lanfear et al., 2012) followed by ML tree inference and 1000 ultrafast bootstrap replicates (Hoang et al., 2018). Bayesian analyses were performed using MrBayes v3.2.7 (Ronquist et al., 2012). We used Akaike Information Criterion (AIC) in JMODELTEST v2.1.7 (Santorum et al., 2014) to determine the best-fitting model of molecular evolution was GTR+I+G (**Table 3**). Each Markov chain Monte Carlo (MCMC) run was conducted for 50 million generations sampling every 1000 generations. The first 25% of trees were discarded as burn-in, and the remaining trees were used to construct a consensus tree to estimate the posterior probabilities (PPs). We used Tracer v1.7.2 (Rambaut et al., 2018) to evaluate convergence and effective sample size (ESS > 200). All phylogenetic trees were visually analyzed using FigTree v1.4.4 (http://tree.bio.ed.ac.uk/software/figtree).

**Table 3:** Characteristics and models selected for different datasets in ML and BI analysis.

Datasets	Number of taxa	Number of sites	Number of variable/Parsimony informative sites	Best fit Model	Model in ML	Model in BI
Whole plastid genomes	77	189903	38835/27241	GTR+I+G	GTR + G	GTR+I+G
Coding gene	77	71055	10853/6929	GTR+I+G	GTR + G	GTR+I+G
Non-coding regions	77	80810	19975/12736	GTR+I+G	GTR + G	GTR+I+G
LSC	77	108001	23357/15551	GTR+I+G	GTR+G	GTR+I+G
SSC	77	26380	11426/9689	GTR+G	GTR+G	GTR+G
IRb	77	29466	1891/956	GTR+I+G	GTR+G	GTR+I+G
NON-IRa	77	161421	36867/26333	GTR+I+G	GTR+G	GTR+I+G

### Divergence time estimation

We performed a dated phylogenetic analysis using BEAST v2.6.6 (Drummond et al., 2012) to investigate the historical biogeography of Arecaceae. The BEAST analysis used a Yule speciation prior and an uncorrelated log normal (UCLN) relaxed clock to estimate the divergence time. The best performing model of molecular evolution was selected as the GTR+I+G model according to AIC selected by MrModelTest v2.4 (Posada, 2008). The age of the crown node of Arecaceae was calibrated using fossil calibration points and secondary calibration points. According to Khan et al. (2020) the well-preserved petrified palm stem fossils, *Palmoxylon ceroxyloides*, dating to 66-65 Ma from the Deccan Traps, was identified as the oldest stems of Ceroxyloideae in the fossil record. Therefore, we set a log-normal prior for the stem of Ceroxyloideae clade with a fossil crown age of 65.0 Ma. Based on the rich Arecaceae fruit fossil record of Matsunaga and Smith (2021), they suggest that some fossils were suitable as node calibrations. Here, we used two of these fossils as node calibrations. We used a log-normal distribution to set the crown age of Trachycarpeae to 62.0 Ma and the crown group age of Areceae to 47.0 Ma. Based on the phylogeny estimated by Li et al. (2019), the crown group age of Arecales was constrained to 96.2 Ma, setting a secondary calibration point of the crown age of Arecaceae+Asparagaceae branch to 96.2 Ma using a normal prior with a mean=96.2 Ma and stdev=5. The MCMC chain length set at 9 x10^8^ generations sampling every 1000 generations. We used Tracer v1.7.2 (Rambaut et al., 2018) to evaluate convergence and effective sample size (ESS > 200), while discarding the first 10% of trees. LogCombiner v2.6.6 (Drummond et al., 2012) was used to eliminate burn-in trees and merge tree files from nine runs, and the first 25% of trees were discarded as burn-in. TreeAnnotator v2.6.6 (Drummond et al., 2012) was used to generate a maximum clade credibility (MCC) tree. The final inferred tree was visually analyzed using FigTree v1.4.4 (http://tree.bio.ed.ac.uk/software/figtree/) showing the mean divergence time estimates with 95% maximum posterior density (HPD) intervals.

### Morphological evolution analysis of Arecaceae

This study analyzed the historical reconstruction of ancestral morphological characters of key traits in Arecaceae based on the topology of the whole chloroplast genome for ML analysis, using the “Trace character history” option in Mesquite v3.51, and the Markov k-state reference model (Maddison, 2008). The data for the morphological characteristics of Arecaceae were mainly obtained from our field observations of the samples and the literature (Moore and Uhl, 1982; Dransfield et al., 2008; Horn et al., 2009; Matsunaga and Smith, 2021). The morphological traits are shown in **Table 5**. Associated codes for species-specific traits that were scored include: A) Number of stamens: (0) 3; (1) 6; (2) 10-40; (3) 40+; B) Number of seeds: (0) 1; (1) 1-2; (2) 1-3; (3) 3; (4) 4-10; C) Pericarp type: (0) Smooth; (1) Rough; (2) Spiny; (3) Imbricate scales; D) Plant type: (0) Monoecious; (1) Dioecious.

## Results

### Structural features of chloroplast genomes

**Figure 2:**
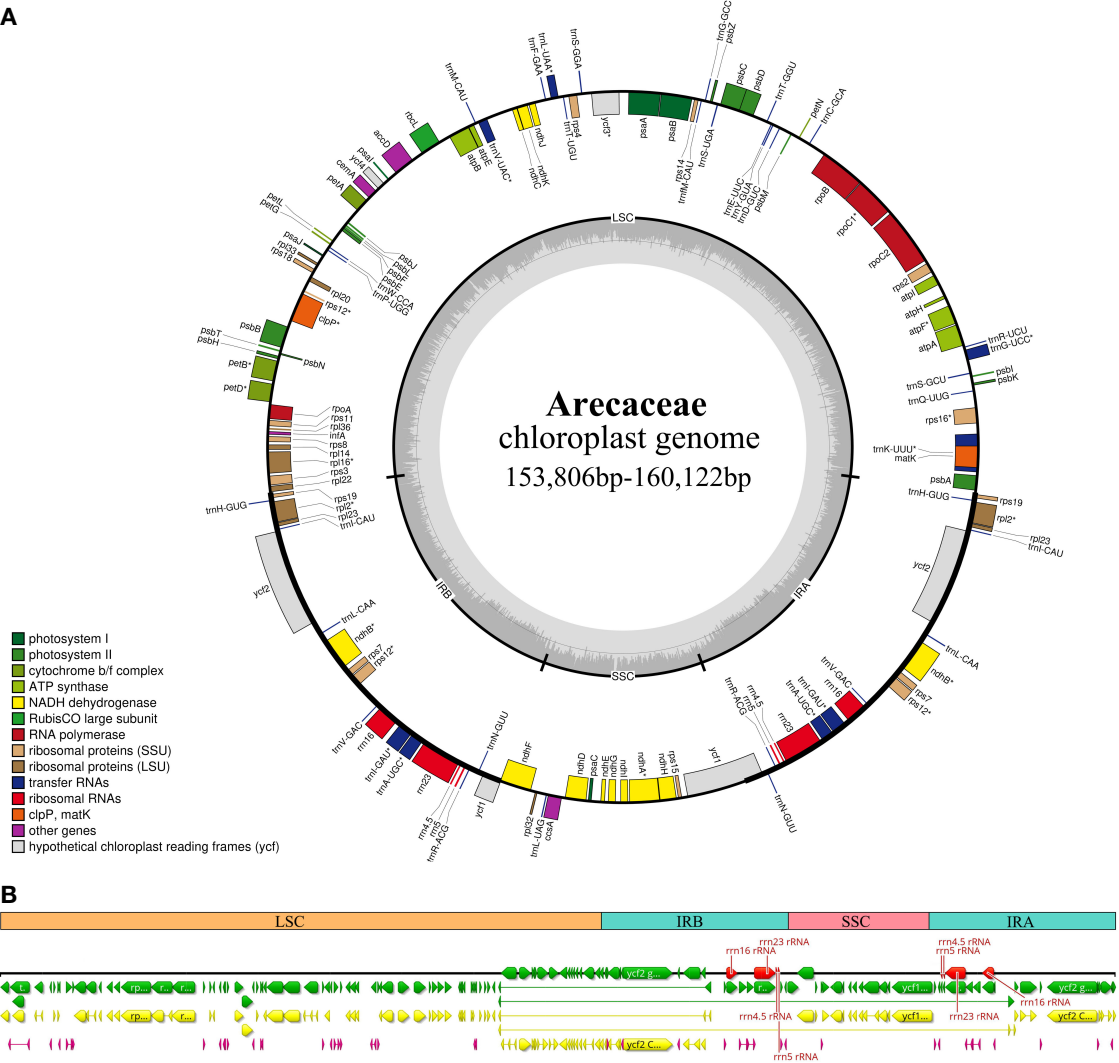
Chloroplast genome map of Arecaceae. **(A)** Circular form. Genes inside the outer circles are transcribed clockwise and those outside the circles are transcribed counterclockwise. The gray variation area in the inner circle indicates the GC content of the chloroplast genome. Different colors indicate different functional genes. **(B)** Linear form. Different colors indicate different functional genes, green indicates Genes, yellow indicates Protein Coding Genes, red indicates rRNA genes, and purple indicates tRNA genes.

In this study, we investigated 74 chloroplast genomes in Arecaceae, covering five subfamilies: Arecoideae (24 species in five tribes), Calamoideae (11 species in three tribes), Ceroxyloideae (two species in two tribes), Coryphoideae (36 species in eight tribes) and Nypoideae (one species in one tribe). The results show that all 74 chloroplast genomes display the typical tetrad structure of angiosperms: a LSC region (82,900–87,639 bp), a SSC region (13,768–18,380 bp), and a pair of IR regions (26,007–28,228 bp) (**Figure 2**; **Table 2**). The length of the 74 plastomes range from 153,806 to 160,122 bp, with a size difference of 6,316 bp. The differences in the LSC, SSC and IR regions span 4,739 bp, 4,612 bp and 2,221 bp, respectively. The plastome length of the 24 species of Arecoideae range from 154,048 bp (*Butia eriospatha*, NC_058633.1) to 159,237 bp (*Euterpe oleracea*, OL674119), while the plastomes of the 11 species of Calamoideae range from 154,031 bp (*Eremospatha macrocarpa*, NC_029964.1) to 158,029 bp (*Calamus jenkinsianus*, OL674138) in length. The plastome length of the two species of Ceroxyloideae are 157,829 bp (*Pseudophoenix vinifera*, NC_020364.1) and 159,075 bp (*Phytelephas aequatorialis*, NC_029957.1). The length of the 36 species of Coryphoideae range from 153,806 bp (*Chuniophoenix nana*, NC_029966.1) to 160,122 bp (*Latania lontaroides*, OL674141), and the length of the one species of Nypoideae is 158,391 bp (*Nypa fruticans*, NC_029958.1). All Arecaceae plastomes encode a total of 130-137 genes, of which 112-117 genes (76-82 unique protein-coding genes, 29-32 tRNA genes, and 4 rRNA genes) are located in the single copy regions and 18-20 genes are duplicated in the IR regions. The total GC content of the plastomes are highly similar, ranging from 36.9-37.7%, and the average GC content of the plastome was 37.3%, while the GC content in the LSC, SSC and IR regions are 34.8-35.9%, 31.0-31.6%, and 42.3-42.8%, respectively (**Tables 2**, **4**). In addition, multiple genome alignments were performed on 74 Arecaceae plants to determine whether Arecaceae plastomes were rearranged. The Mauve alignment results are detailed in the attachment (**Figure S1**).

**Table 4:** Average length and G+C content for complete plastomes of the each subfamilies in Arecaceae.

Subfamily	Number of species	Average length (bp) and Average GC content (%)										
		Total			LSC			SSC			IR	
		Length	G+C		Length	G+C		Length	G+C		Length	G+C
Arecoideae	24	157489	37.36		85943	35.41		17572	31.04		26980	42.54
Calamoideae	11	156664	37.45		85256	35.45		17154	31.35		27099	42.40
Ceroxyloideae	2	158452	37.25		86478	35.35		17613	30.70		27103	42.50
Coryphoideae	36	158445	37.22		86383	35.26		17729	30.80		27164	42.43
Nypoideae	1	158391	37.20		86496	35.20		17676	30.70		27179	42.40

### Comparative genomic analysis and SC/IR boundary comparisons

**Figure 3:**
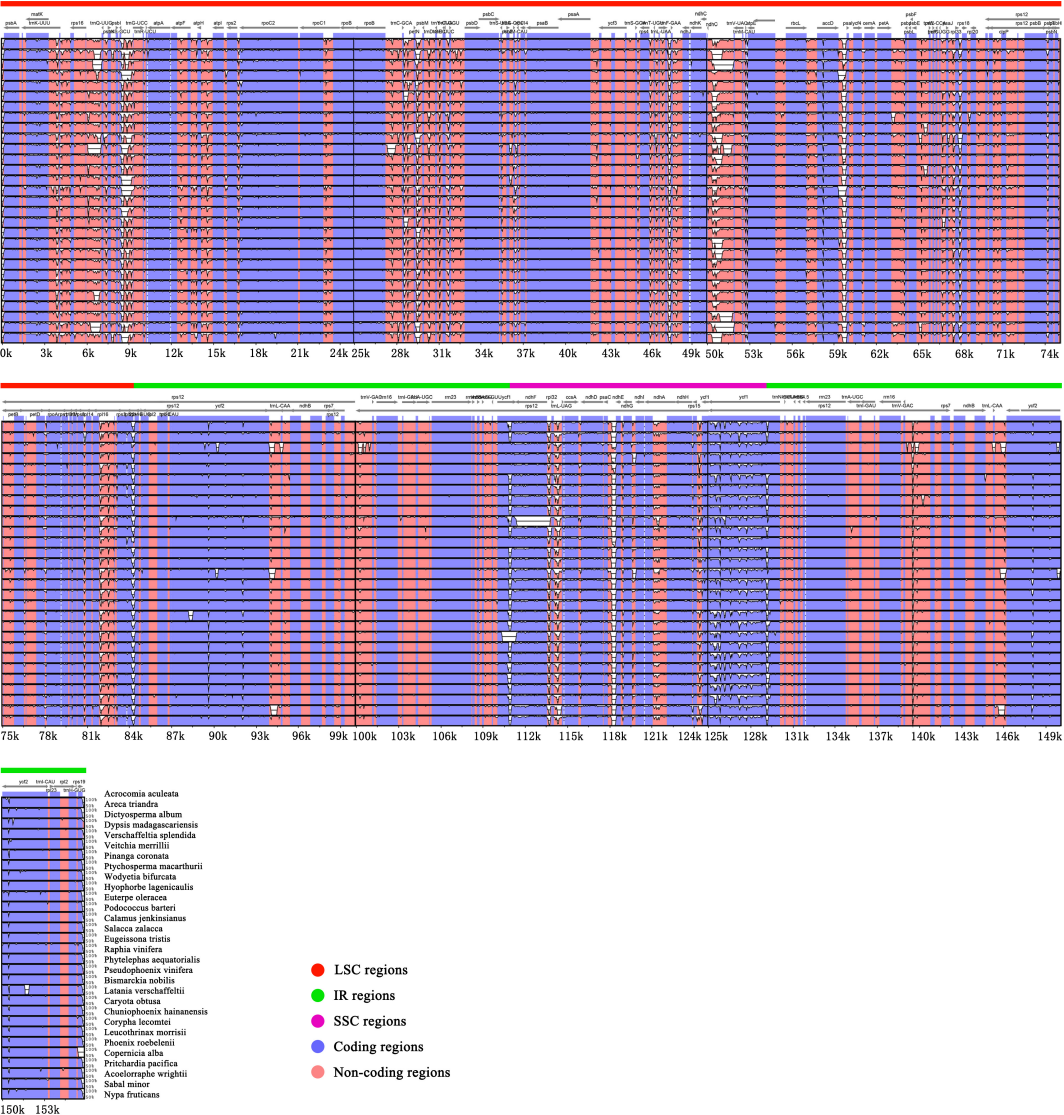
Comparative visualization of chloroplast genome sequence differences in Arecaceae. The protein coding and non-coding regions correspond to blue and red. The x-axis uses *Acrocomia aculeata* as reference sequence and y-axis indicates the percentage of sequence identity (50%-100%).

In this study, we used mVISTA to analyze the sequence differences of 30 representative chloroplast genomes in Arecaceae, using *Acrocomia aculeata* as a reference. The alignments show that the plastomes of the 30 Arecaceae species have few differences (**Figure 3**). The sequence identity of the coding regions are higher than that of non-coding regions, and the sequence identity of the IR region is higher than that of the SC region.

**Figure 4:**
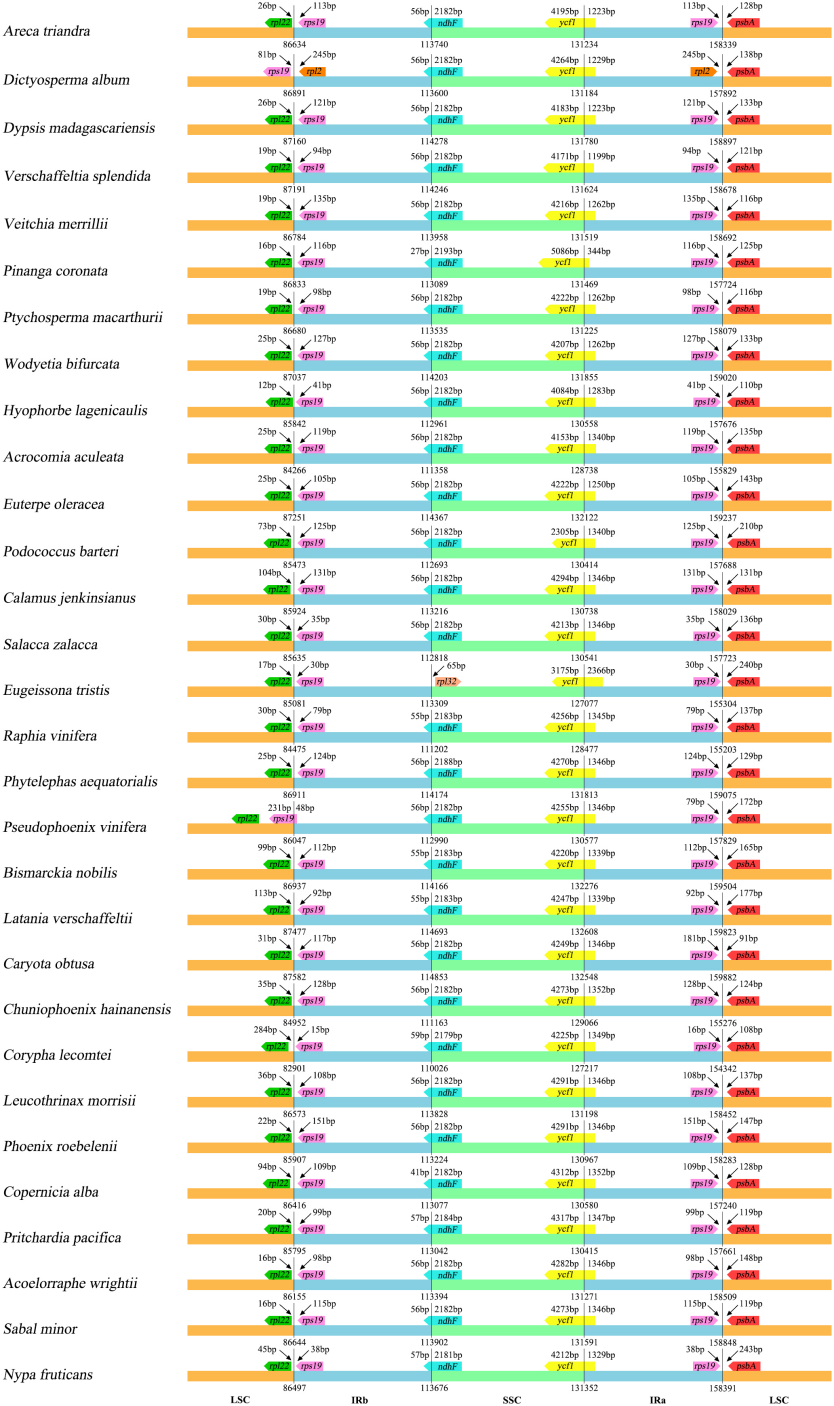
Comparison of the SC/IR junctions of 30 representative chloroplast genomes in Arecaceae.

The results show the differences between the SC and IR boundary regions are structurally similar across the 30 species, and the boundary genes between SC/IR regions are stable. The same genes had the same relative position at the junction of SC/IR regions, with a few exceptions (**Figure 4**). At the LSC/IRb junction, *rpl22* and *rps19* were detected in 28 chloroplast genomes (*rpl22* gene is located in the LSC and *rps19* gene is located in the IRb), while the LSC/IRb junction of *Dictyosperma album* was detected as *rps19* and *rpl2* (*rps19* gene is located in the LSC and *rpl2* is located in the IRb), and *Pseudophoenix vinifera* showed that *rps19* straddles the LSC/IR boundary, with a length of 48 bp in the IRb. Except for *Eugeissona tristis*, the gene detected at the IRb/SSC boundary was *rpl32* (the gene was located in the SSC), and *ndhF* was detected across the IRb/SSC boundary in other species. *ycf1* was detected at the SSC/IRa boundary in all 30 species. For one species, *Dictyosperma album*, *rpl2* and *psbA* (*rpl2* is located in the IRa) were detected at the IRa/LSC boundary, while *rps19* and *psbA* were detected in all other species.

### Phylogenetic results

**Figure 5:**
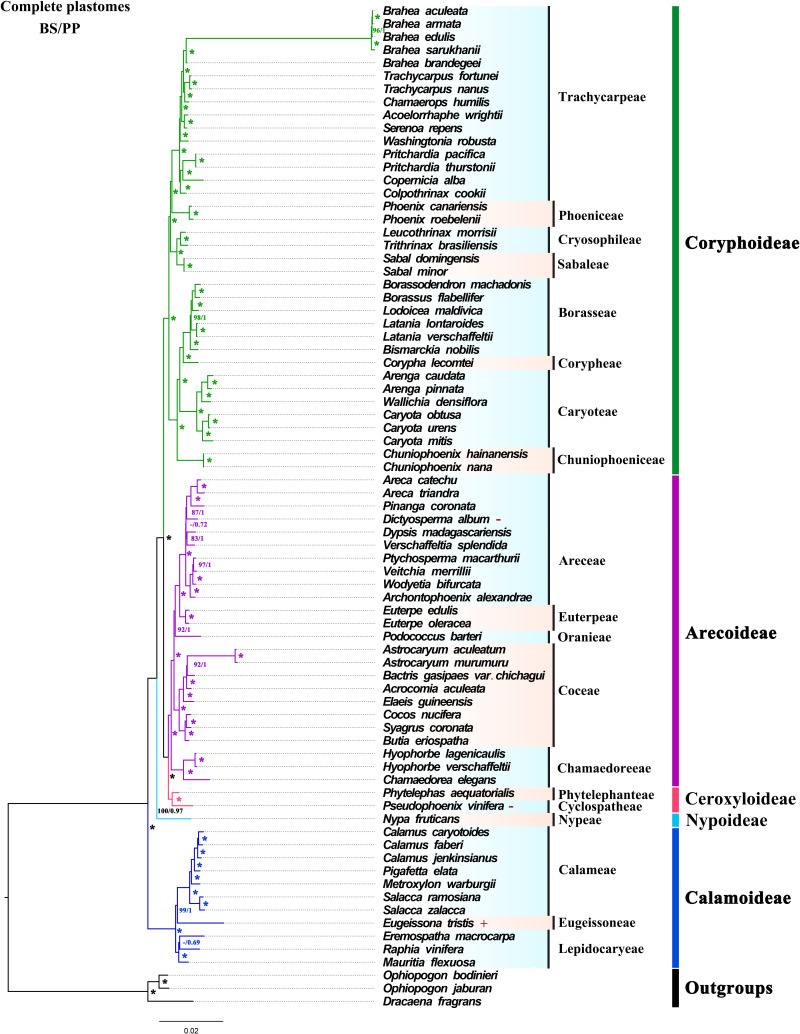
ML and BI trees were constructed based on the whole chloroplast genome dataset. “*” indicate support values of 100%/1.0, numbers near the nodes indicate 60% and 0.6 or more support obtained from the analysis, and “-” is used when both support values are less than 60% or 0.6. Different colors represent different clades of subfamilies. The “+” presented to the right of the species indicates that the species expands at the IR/SC boundary, and “-” indicates that the species contracts at the IR/SC boundary.

Based on 74 whole plastomes, the topology generated by ML and BI phylogenetic inference were consistent with full support for each node [ML bootstrap (BS) = 100%, Bayesian posterior probabilities (PP) = 1] (**Figure 5**). At the subfamily level, the phylogenetic tree fully showed that the five subfamilies were all monophyletic, and the relationships between genera had high support. In the phylogenetic trees, Ceroxyloideae and Arecoideae are sister groups, and Coryphoideae was confirmed as the sister group of Ceroxyloideae + Arecoideae (BS/PP = 100/1). Nypoideae was identified as the sister of [Coryphoideae+ [Ceroxyloideae +Arecoideae]] with strong support, while Calamoideae was identified as the sister to all other Arecaceae with full support (BS/PP = 100/1). The phylogenetic trees (ML/BI) inferred using the LSC, No-IRa, protein-coding sequence (CDS) and non-protein-coding sequence (Non-CDS) (**Figures S2, S5, S6, S7**), showed relationships at the subfamily level consistent with those using whole plastomes. However, using just the SSC region, a separate clade of Coryphoideae and the clade of [Nypoideae+[Coryphoideae+[Ceroxyloideae+Arecoideae]]] formed a sister relationship, and Coryphoideae was not monophyletic (**Figure S3**). In the phylogenetic tree reconstructed with the IRb region, Ceroxyloideae and Coryphoideae were found to be sisters, while Arecoideae was the sister group of Ceroxyloideae+Coryphoideae and was not monophyletic with high support (**Figure S4**).

### Divergence time estimation of Arecaceae

**Figure 6:**
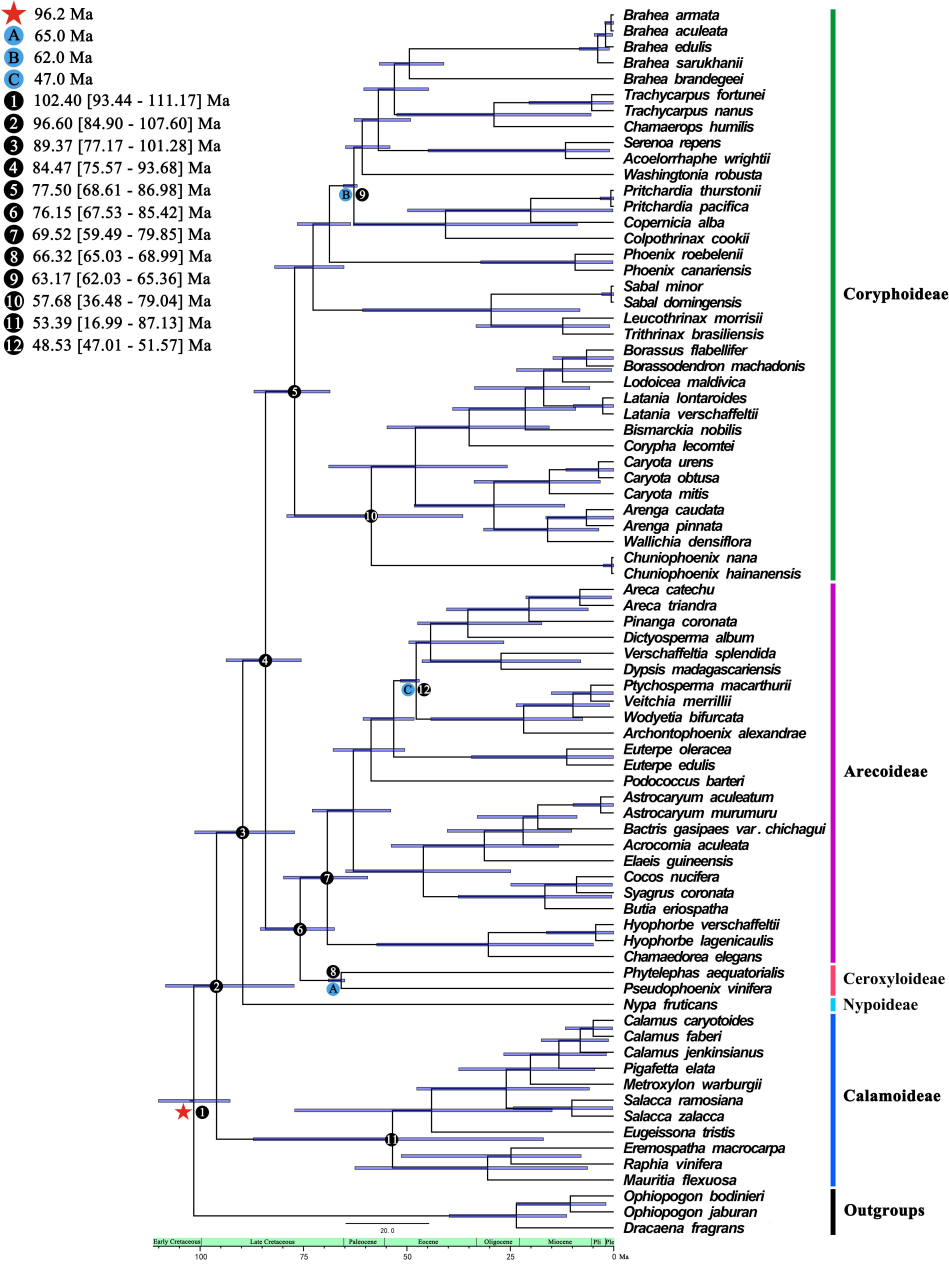
Species divergence times based on whole chloroplast genome datasets analyzed from BEAST. A, B, and C are calibration points, respectively, and star shape are secondary calibration points. Numbers 1-12 represent the estimated mean divergence times of the main divergence events and 95% highest posterior densities.

We estimated the divergence time using the 74 whole plastomes of Arecaceae using BEAST (**Figure 6**). The crown group age of Arecaceae was 96.60 Ma (95% HPD = 84.90–107.60 Ma), and the stem age was 102.40 Ma (95% HPD = 93.44–111.17 Ma), which corresponds to the Early Cretaceous. The clades of Nypoideae and [[Ceroxyloideae+Arecoideae]+Coryphoideae] (crown group age: 84.47 Ma, 95% HPD = 75.57–93.68 Ma) diverged 89.37 Ma during the Late Cretaceous. Approximately 84.47 Ma (95% HPD = 75.57–93.68 Ma) the Ceroxyloideae and Arecoideae clade diverged from the Coryphoideae, and the crown group of Coryphoideae was dated to 77.50 Ma (95% HPD = 68.61–86.98 Ma). The crown group of Ceroxyloideae (crown group age: 66.32 Ma, 95% HPD = 65.03–68.99 Ma) and Arecoideae (crown group age: 69.52 Ma, 95% HPD = 59.49–79.85 Ma) diverged 76.15 Ma (95% HPD = 67.53–85.42 Ma). In addition, the Calamoideae crown group diverged 53.39 Ma (95% HPD = 16.99–87.13 Ma) during the Eocene.

### Ancestral trait reconstruction

**Figure 7:**
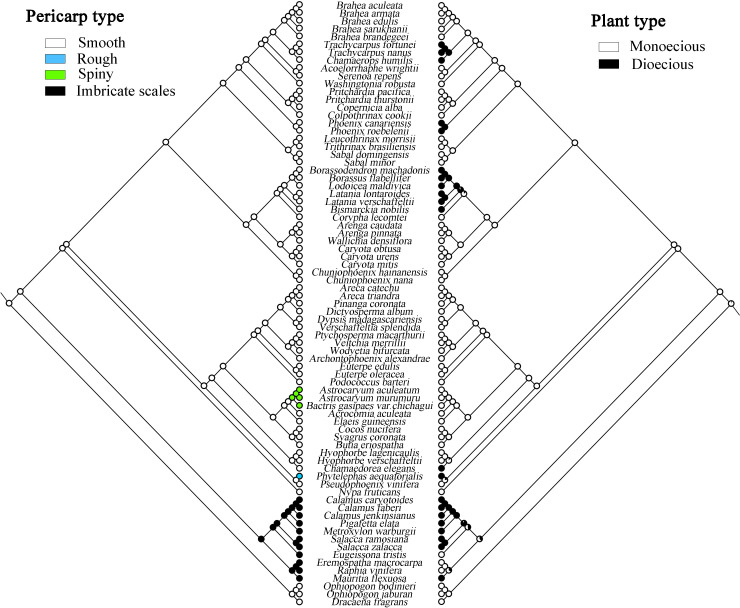
Maximum likelihood analysis of morphological traits evolution in Arecaceae based on the chloroplast data set. Left, Pericarp type; Right, Plant type.

The evolutionary analysis of species morphology in Arecaceae shows that some traits evolved *via* convergent evolution. For example, the trait of pericarp type reflects this phenomenon (**Figure 7**). For this trait, we infer that the ancestral fruit pericarp trait of Arecaceae plants was smooth. The fruit pericarp of species in Calamoideae were all imbricate scales, whereas species in Ceroxyloideae have evolved to have a rough fruit pericarp, and plants of *Phytelephas* in the tribe Phytelephanteae appear to have a rough acute-warty pericarp. In Arecoideae, only species of *Astrocaryum* and *Bactris* in the tribe Coceae have evolved a spiny pericarp, while all other species retain the ancestral trait. In addition, in the Coryphoideae all species retain the original smooth pericarp of the ancestral form. In terms of plant types, the ancestral plant type of Arecaceae was monoecious, which then evolved into a dioecious plant (**Figure 7**). Except for the species of tribe Eugeissoneae and *Eremospatha* and *Raphia* in tribe Lepidocaryeae, which still maintain the original ancestral form in Calamoideae, other species have evolved into dioecious plants. The species of *Phytelephas* in Ceroxyloideae and species of *Chamaedorea* of tribe Chamaedoreaee in Arecoideae have also evolved into dioecious plants. Similar diversity changes have occurred in Coryphoideae, for example, the plants of tribe Borasseae and Phoenixe have evolved dioecy, while in the tribe Trachycarpeae, most species still retain the ancestral form during the evolutionary process.

**Figure 8:**
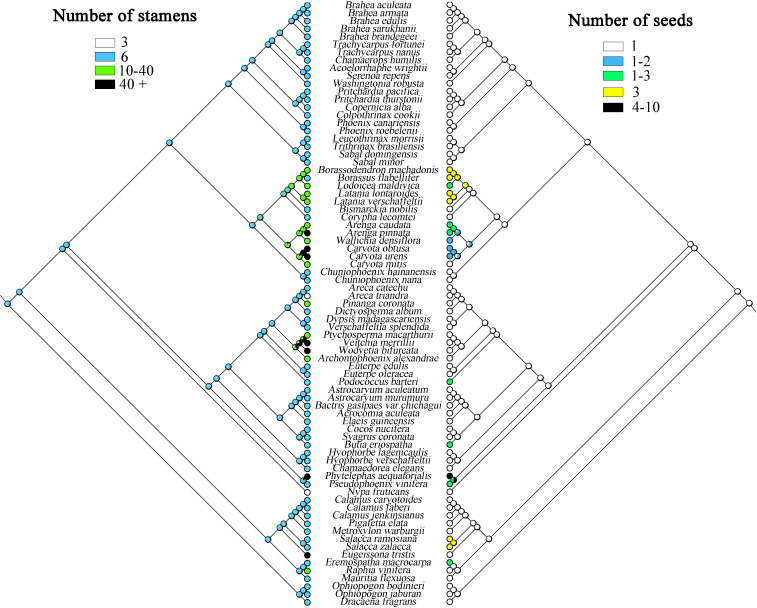
Maximum likelihood analysis of morphological traits evolution in Arecaceae based on the chloroplast data set. Left, Number of stamens; Right, Number of seeds.

The present study infers that species having one seed is the ancestral trait in Arecaceae, while some plants evolved to have more seeds (**Figure 8**), such as in Ceroxyloideae, *Pseudophoenix* of the tribe Cyclospatheae with one to three seeds and even four to 10 seeds in *Phytelephas* of the tribe Phytelephanteae. The number of seeds of species in Calamoideae and Coryphoideae show high diversity, for example *Salacca* in the tribe Calameae of Calamoideae independently evolved three seeds, species of *Eremospatha* in the tribe Lepidocaryeae evolved one to three seeds, while in Coryphoideae some species of the tribe Borasseae evolved independently one to three or three seeds, and in the tribe Caryoteae, seeds ranged from one to two or one to three seeds. In Arecoideae, plants of *Butia* in the tribe Coceae and plants of *Podococcus* in the tribe Oranieae also evolved one to three seeds independently. Otherwise, the number of seeds in the other Arecaceae species has largely maintained the ancestral form. We also found that the evolution of the number of stamens was complex and infer that having six stamens was the ancestral state of Arecaceae, with multiple instances of evolving a greater number of stamens (**Figure 8**) **(** **Table 5**). In the Calamoideae, only plants of *Eugeissona* in the tribe Eugeissoneae and plants of *Raphia* in the tribe Lepidocaryeae have changed in the number of stamens. Species of *Phytelephas* of the tribe Phytelephanteae in Ceroxyloideae have a large disparity in the number of stamens, ranging from dozens to hundreds. In the Arecoideae only species of the tribe Areceae have evolved diversity, with numbers varying by dozens, while *Areca triandra* have undergone degeneration in stamen number, with numbers reduced to three, and the same degeneration occurring in *Nypa fruticans* of Nypoideae, with stamen numbers also reduced to three. The remaining tribes in Coryphoideae retain their ancestral traits, in addition to the evolution of stamens in species of two tribes, the Borasseae and the Caryoteae. Overall, most plants have undergone long-term evolution from their ancestral forms.

**Table 5:** Statistics of morphological characteristics of Arecaceae.

Subfamily	Tribe	Species name	Number of stamens (Average of multiple stamens)	Number of seeds	Pericarp type	Plant type
Arecoideae	Areceae	*Archontophoenix alexandrae*	13	1	Smooth	Monoecious
Arecoideae	Areceae	*Areca catechu*	6	1	Smooth	Monoecious
Arecoideae	Areceae	*Areca triandra*	3	1	Smooth	Monoecious
Arecoideae	Areceae	*Dictyosperma album*	6	1	Smooth	Monoecious
Arecoideae	Areceae	*Dypsis madagascariensis*	6	1	Smooth	Monoecious
Arecoideae	Areceae	*Verschaffeltia splendida*	6	1	Smooth	Monoecious
Arecoideae	Areceae	*Pinanga coronata*	40	1	Smooth	Monoecious
Arecoideae	Areceae	*Ptychosperma macarthurii*	33	1	Smooth	Monoecious
Arecoideae	Areceae	*Veitchia merrillii*	53	1	Smooth	Monoecious
Arecoideae	Areceae	*Wodyetia bifurcata*	66	1	Smooth	Monoecious
Arecoideae	Chamaedoreeae	*Chamaedorea elegans*	6	1	Smooth	Dioecious
Arecoideae	Chamaedoreeae	*Hyophorbe lagenicaulis*	6	1	Smooth	Monoecious
Arecoideae	Chamaedoreeae	*Hyophorbe verschaffeltii*	6	1	Smooth	Monoecious
Arecoideae	Coceae	*Acrocomia aculeata*	6	1	Smooth	Monoecious
Arecoideae	Coceae	*Astrocaryum aculeatum*	6	1	Spiny	Monoecious
Arecoideae	Coceae	*Astrocaryum murumuru*	6	1	Spiny	Monoecious
Arecoideae	Coceae	*Cocos nucifera*	6	1	Smooth	Monoecious
Arecoideae	Coceae	*Elaeis guineensis*	6	1	Smooth	Monoecious
Arecoideae	Coceae	*Syagrus coronata*	6	1	Smooth	Monoecious
Arecoideae	Coceae	*Butia eriospatha*	6	1-3	Smooth	Monoecious
Arecoideae	Coceae	*Bactris gasipaes* var. *chichagui*	6	1	Spiny	Monoecious
Arecoideae	Euterpeae	*Euterpe edulis*	6	1	Smooth	Monoecious
Arecoideae	Euterpeae	*Euterpe oleracea*	6	1	Smooth	Monoecious
Arecoideae	Oranieae	*Podococcus barteri*	6	1-3	Smooth	Monoecious
Calamoideae	Calameae	*Calamus faberi*	6	1	Imbricate scales	Dioecious
Calamoideae	Calameae	*Calamus jenkinsianus*	6	1	Imbricate scales	Dioecious
Calamoideae	Calameae	*Calamus caryotoides*	6	1	Imbricate scales	Dioecious
Calamoideae	Calameae	*Metroxylon warburgii*	6	1	Imbricate scales	Dioecious
Calamoideae	Calameae	*Pigafetta elata*	6	1	Imbricate scales	Dioecious
Calamoideae	Calameae	*Salacca ramosiana*	6	3	Imbricate scales	Dioecious
Calamoideae	Calameae	*Salacca zalacca*	6	3	Imbricate scales	Dioecious
Calamoideae	Eugeissoneae	*Eugeissona tristis*	45	1	Imbricate scales	Monoecious
Calamoideae	Lepidocaryeae	*Eremospatha macrocarpa*	6	1-3	Imbricate scales	Monoecious
Calamoideae	Lepidocaryeae	*Mauritia flexuosa*	6	1	Imbricate scales	Dioecious
Calamoideae	Lepidocaryeae	*Raphia vinifera*	18	1	Imbricate scales	Monoecious
Ceroxyloideae	Phytelephanteae	*Phytelephas aequatorialis*	468	4-10	Rough	Dioecious
Ceroxyloideae	Cyclospatheae	*Pseudophoenix vinifera*	6	1-3	Smooth	Monoecious
Coryphoideae	Borasseae	*Bismarckia nobilis*	6	1	Smooth	Dioecious
Coryphoideae	Borasseae	*Borassodendron machadonis*	10	3	Smooth	Dioecious
Coryphoideae	Borasseae	*Borassus flabellifer*	6	3	Smooth	Dioecious
Coryphoideae	Borasseae	*Latania lontaroides*	23	3	Smooth	Dioecious
Coryphoideae	Borasseae	*Latania verschaffeltii*	24	3	Smooth	Dioecious
Coryphoideae	Borasseae	*Lodoicea maldivica*	25	1-3	Smooth	Dioecious
Coryphoideae	Caryoteae	*Arenga caudata*	25	1-3	Smooth	Monoecious
Coryphoideae	Caryoteae	*Arenga pinnata*	90	1-3	Smooth	Monoecious
Coryphoideae	Caryoteae	*Caryota mitis*	18	1	Smooth	Monoecious
Coryphoideae	Caryoteae	*Caryota obtusa*	53	1-2	Smooth	Monoecious
Coryphoideae	Caryoteae	*Caryota urens*	65	1-2	Smooth	Monoecious
Coryphoideae	Caryoteae	*Wallichia densiflora*	10	1-2	Smooth	Monoecious
Coryphoideae	Chuniophoeniceae	*Chuniophoenix hainanensis*	6	1	Smooth	Monoecious
Coryphoideae	Chuniophoeniceae	*Chuniophoenix nana*	6	1	Smooth	Monoecious
Coryphoideae	Corypheae	*Corypha lecomtei*	6	1	Smooth	Monoecious
Coryphoideae	Cryosophileae	*Leucothrinax morrisii*	6	1	Smooth	Monoecious
Coryphoideae	Cryosophileae	*Trithrinax brasiliensis*	6	1	Smooth	Monoecious
Coryphoideae	Phoeniceae	*Phoenix canariensis*	6	1	Smooth	Dioecious
Coryphoideae	Phoeniceae	*Phoenix roebelenii*	6	1	Smooth	Dioecious
Coryphoideae	Trachycarpeae	*Brahea aculeata*	6	1	Smooth	Monoecious
Coryphoideae	Trachycarpeae	*Brahea armata*	6	1	Smooth	Monoecious
Coryphoideae	Trachycarpeae	*Brahea brandegeei*	6	1	Smooth	Monoecious
Coryphoideae	Trachycarpeae	*Brahea edulis*	6	1	Smooth	Monoecious
Coryphoideae	Trachycarpeae	*Brahea sarukhanii*	6	1	Smooth	Monoecious
Coryphoideae	Trachycarpeae	*Colpothrinax cookii*	6	1	Smooth	Monoecious
Coryphoideae	Trachycarpeae	*Copernicia alba*	6	1	Smooth	Monoecious
Coryphoideae	Trachycarpeae	*Pritchardia pacifica*	6	1	Smooth	Monoecious
Coryphoideae	Trachycarpeae	*Pritchardia thurstonii*	6	1	Smooth	Monoecious
Coryphoideae	Trachycarpeae	*Acoelorraphe wrightii*	6	1	Smooth	Monoecious
Coryphoideae	Trachycarpeae	*Serenoa repens*	6	1	Smooth	Monoecious
Coryphoideae	Trachycarpeae	*Chamaerops humilis*	6	1	Smooth	Dioecious
Coryphoideae	Trachycarpeae	*Trachycarpus fortunei*	6	1	Smooth	Dioecious
Coryphoideae	Trachycarpeae	*Trachycarpus nanus*	6	1	Smooth	Dioecious
Coryphoideae	Trachycarpeae	*Washingtonia robusta*	6	1	Smooth	Monoecious
Coryphoideae	Sabaleae	*Sabal domingensis*	6	1	Smooth	Monoecious
Coryphoideae	Sabaleae	*Sabal minor*	6	1	Smooth	Monoecious
Nypoideae	Nypeae	*Nypa fruticans*	3	1	Smooth	Monoecious
Ruscoideae	Ophiopogoneae	*Ophiopogon bodinieri*	6	1	Smooth	Monoecious
Ruscoideae	Ophiopogoneae	*Ophiopogon jaburan*	6	1	Smooth	Monoecious
Ruscoideae	Dracaeneae	*Dracaena fragrans*	6	1	Smooth	Monoecious

## Discussion

### Plastome genome structure comparisons and sequence difference analysis

According to the comparative results of chloroplast genome structure (**Figure 2**; **Table 2**), the size of the 74 chloroplast genomes of Arecaceae range from 153,806-160,122 bp, of which LSC length was 82,900-87,639 bp, the SSC was 13,768-18,380 bp, and the IR was 26,007-28,228 bp. There were 76-82 unique protein-coding genes, 29-32 tRNA genes and four rRNA genes. The structural features of Arecaceae plastomes were similar to those of most other angiosperms (Palmer, 1991; Raubeson and Jansen, 2005; Chumley et al., 2006; Huang et al., 2016). Studies had shown that the chloroplast genome of land plants is a circular double-stranded DNA molecule ranging in size from 107-218 kb, which can vary from species to species (Daniell et al., 2016). In angiosperms, the chloroplast genome is usually between 120-160 kb in size, with sequences of the LSC between 80-90 kb, the SSC between 16-27 kb and the IR between 20-28 kb (Palmer and Delwiche, 1998; Raubeson and Jansen, 2005; Chumley et al., 2006; Zhang et al., 2012; Serrano et al., 2013). This indicates that the Arecaceae chloroplast genome size was within the normal range of angiosperm chloroplast genome size. According to our results, both the largest chloroplast genome (160,122 bp) and the smallest chloroplast genome (153,806 bp) were in Coryphoideae. The differences in chloroplast genome size among different subfamilies were primarily due to differences in the length of the IR region. Due to the contraction and expansion of IR region, there were differences between IR/SC boundaries of plastid genome (Kim and Lee, 2004; Chang et al., 2006; Raubeson et al., 2007). These changes may lead to gene loss or gain (Wicke et al., 2011; Wang et al., 2018), which are usually the main cause of changes in the size of the plastome (Kim and Lee, 2004; Wang and Messing, 2011; Zhang et al., 2016). In our results (**Figure 4**), the IR regions of *Dictyosperma album* in Arecoideae and *Pseudophoenix vinifera* in Ceroxyloideae were both contracted at the IRb/LSC boundary; the *rps19* gene of *Dictyosperma album* was complete in the LSC region, while the length of *rps19* in *Pseudophoenix vinifera* was 231 bp in the LSC region and 48 bp in the IRb region. The IR region of *Eugeissona tristis* in Calamoideae was expanded at the IRa/SSC boundary, and *ycf1* expanded to 2366 bp in the IRa region, resulting in the loss of *ndhF*, making the IRb/SSC boundary *rpl32*. The phenomenon of expansion or contraction of the IR region (Chumley et al., 2006; Wang et al., 2008; Guisinger et al., 2010; Yang et al., 2010; Sun et al., 2013) and gene loss (Wicke et al., 2011; Barrett et al., 2014) had also been found in previous studies.

To analyze the different levels of gene sequences, this study used mVISTA to draw sequence identity plots (**Figure 3**). The results showed that the non-coding regions showed a higher level of divergence than the coding regions. Most of the sequence divergence was concentrated in the non-coding regions which were less conserved than the coding regions, similar to most chloroplast genomes of angiosperms (Perry and Wolfe, 2002; Huang et al., 2014; Zhang et al., 2016). In addition, the diversity variation in the IR region was smaller than that in the SC regions (LSC and SSC). The small variation in the IR region was primarily due to the duplication correction caused by gene conversion between IR sequences (Khakhlova and Bock, 2006). In conclusion, coding region and IR region showed higher conservation than non-coding region and SC region.

### Phylogenetic relationships of Arecaceae

Compared to earlier phylogenetic studies of Arecaceae (Hahn, 2002a; Asmussen et al., 2006; Eiserhardt et al., 2011; Faurby et al., 2016), this study was the first to use plastome sequences for segmentation and data partitioning to analyze the phylogenetic relationship of Arecaceae. Based on the complete chloroplast genome, phylogenetic analysis of the seven datasets was performed using ML and BI analysis. The topological structure based on ML and BI analysis were highly consistent in each data set. Except for the SSC and IRb datasets, the phylogenetic relationships inferred from the other five datasets (whole chloroplast genome, LSC, No-IRa, CDS and Non-CDS) were consistent by strongly supporting the five subfamilies were monophyletic and the relationships within each subfamily (BS/PP = 100/1). Our results show similar relationships as in most other studies of Arecaceae (Asmussen et al., 2006, which relied on plastid DNA; Baker et al., 2009, which relied on plastid, nuclear ribosomal, and low-copy nuclear DNA; Faurby et al., 2016, which relied on morphological and genetic data) and strongly support a sister relationship between Ceroxyloideae and Arecoideae (BS/PP = 100/1). Furthermore, the relationships among the tribes/genera were well-supported based on the phylogenetic relationships of the whole chloroplast genome. However, the phylogenetic positions of tribes differ in different studies. In Arecoideae, the intertribal relationships in this study were consistent with those in Baker et al. (2009) and Faurby et al. (2016) (Chumaedoreae + [Coceae + [Oranieae + [Areceae + Euterpeae]]]), whereas Comer et al. (2015; 2016) inferred different relationships (Chumaedoreeae+[Oranieae+[Coceae+[Areceae+Euterpeae]]]). In Coryphoideae, Sabaleae+Cryosophileae and Trachycarpeae+Phoeniceae were sisters, while in Faurby et al. (2016) Sabaleae and [Cryosophileae+[Trachycarpeae+Phoeniceae]] were sisters, and in Baker et al. (2009) Phoeniceae was the sister group of [Trachycarpeae+[Sabalaee+Cryosophileae]]. The different studies cited above all used different data and methods, while the sampling was also not the same, so unsurprisingly different topologies were generated. In addition, the all-evidence species-level supertree of Faurby et al. (2016) and the complete genus-level supermatrix tree of Baker et al. (2009) showed great differences in the phylogenetic position of the intergeneric relationships within tribes, with the differences largely due to differences in taxa sampling. Here, this study lacks more species in some of the genera/tribes and cannot fully compare the relationship between all genera/tribes with previous studies. Notably, due to the small number of taxa sampled in this study, the relationships between tribes, especially intergeneric, are still not clear, which is a limitation. Therefore, the evolutionary relationships and division among the various tribes/genera still need further studying with expanded sampling and more molecular data.

### Molecular dating

Correct phylogenetic relationships and estimates of divergence times are important for evolutionary studies. We selected the complete chloroplast genome dataset to facilitate and optimize the estimation of divergence time. Molecular dating results suggest that the diversification of Arecaceae most likely began 96.60 [84.90-107.60] Ma in the late Cretaceous. We infer that the crown age of Arecaceae (96.60 Ma) was younger than that estimated by Janssen and Bremer (2004; 110 Ma), Onstein et al. (2018; ca 110 Ma), older than that estimated by Mennes et al. (2015; 84-90 Ma) and Givnish et al. (2018; ca 85 Ma), and similar to the results estimated by Li et al. (2019; 96.2 Ma), Matsunaga and Smith (2021; ca 93 Ma), Couvreur et al. (2011; 100 Ma), and Baker and Couvreur (2013; 100 Ma). The differences in divergence time estimates between different studies may be due to factors such as the setting of fossil calibration points, taxon sampling, choice of molecular data, and different operating methods. Compared with other related studies (Janssen and Bremer, 2004; Couvreur et al., 2011; Baker and Couvreur, 2013; Mennes et al., 2015), this study selected two new suitable fossil node calibrations, and a secondary calibration point as the limit of the total root crown group time. Our findings were similar to those of most other studies, with the Arecaceae lineage originating in the Cretaceous and early Paleogene (Janssen and Bremer, 2004; Harley, 2006; Couvreur et al., 2011; Baker and Couvreur, 2013; Givnish et al., 2018; Matsunaga and Smith, 2021). The divergence times of subfamilies except Calamoideae were similar to the results of Couvreur et al., 2011; Baker and Couvreur (2013) (Nypoideae, stem, 93.5 Ma; Coryphoideae, stem, 86.6 Ma; Ceroxyloideae, stem, 78.2 Ma; Arecoideae, crown, 73.6 Ma). The crown age of Calamoideae in these two studies was 80.2 Ma, while the study by Baker and Dransfield (2000) also supported the early fossil record of *Calamus* originating in Gondwana, and Hartwich et al. (2010) found the large palm fossil of late Eocene also suggesting that Calamoideae was distributed early in Gondwana. However, our results (53.39 Ma) were quite different from those studies and therefore need to be interpreted with caution. Different studies run different generations with BEAST, resulting in different crown-group results. Our results infer that Ceroxyloideae originated in Gondwana during the Cretaceous, which was similar to the findings of Khan et al. (2020) which showed that the Ceroxyloideae diverged at the Cretaceous-Paleogene boundary of central India (ca 66-65 Ma) and were present in India about 10-15 million years before the collision between India with Eurasia. After the collision, the group may have dispersed in East Asia, North America, and reached South America during the Miocene. Our dated phylogenetic relationships indicate that the major lineages of Arecaceae diverged during the Late Cretaceous and underwent rapid speciation events from the Paleocene to Eocene, with widespread distributions in the Eocene. The palm radiation primarily occurred in the Early Cenozoic, and with the warming of the climate and the passage of time, the species diversity gradually increased (Daghlian, 1981; Kvaček and Herman, 2004); while the species diversity and distribution range decreased with the cooling in the Oligocene and Late Miocene (Daghlian, 1981; Harley, 2006), suggesting that species diversity changes were related to climate, meanwhile indicating the existence of a subtropical to tropical paleoclimate in the Late Eocene (Hartwich et al., 2010).

### Morphological evolution of species

In this study, we used several traits of Arecaceae, stamen number, seed number, plant type, and pericarp type to reconstruct ancestral traits. The results of reconstructed ancestral traits show that a smooth pericarp is the likely ancestral trait of the family. For this trait, we infer that the smooth fruit pericarp gradually evolved into spiny, imbricate scales, and rough pericarp. There is extensive homogeneity in morphological characters among species of Arecaceae. For example, in Calamoideae, the fruit pericarp is covered with scales, whereas in other subfamilies most of the fruit pericarps are smooth except for a few species. Although there is clear convergent evolution of fruit structure diversity and many traits in Arecaceae, fruit traits can still be strongly taxonomically distinct below the subfamily level (Moore and Uhl, 1982). Similarly, the fruits of Calamoideae are most easily distinguished from other subfamilies. Moore and Uhl (1982) also showed the evolution of fruit development in Arecaceae from fleshy to dry and fibrous fruits. For the trait of plant type, the results indicate that monoecy may be the ancestral trait of the family. In angiosperms, hermaphroditism is considered to be the ancestral state (Endress and Doyle, 2009). Weiblen et al. (2000) inferred that the ancestral trait of monocotyledons was hermaphroditic and concluded that dioecy from hermaphroditism underwent transformations more frequently than that from monoecy, and that reversals from dioecy to monoecy also occured. In addition, the transition model and mechanism from hermaphroditism to dioecy may be through a transient gynodioecious phase. As shown in De Jong et al. (2008) in a model of sex allocation at the flower level, a possible pathway for the transition from hermaphroditism to monoecy is through andromonoecy. Renner and Ricklefs (1995) indicated that dioecy may have evolved from monoecy through different adjustments in flower sex ratios among individual plants. Moore and Uhl (1982) showed that Arecaceae evolved from hermaphroditism to monoecism and later with polygamy or monoecism to dioecism. In Arecoideae, the largest subfamily of Arecaceae, species are overwhelmingly monoecious, while only *Chamaedorea* in the tribe Chamaedoreeae evolved as a dioecious plant. Castaño et al. (2014) considered that dioecy has evolved twice independently from a monoecious ancestor in this tribe, and the genus *Chamaedorea* exhibits high variability in reproductive morphology. In this case, the origin of Arecaceae plants was ambiguous, and Arecaceae was simply rated as a family that has both monoecy and dioecy.

The ancestral state reconstructions indicate that a seed number of one is the ancestral trait in Arecaceae. Seeds are relatively stable during evolution, with only a few evolving to vary from one to three seeds, and even four to 10 seeds in *Phytelephas* of Ceroxyloideae. This occurrence of many seeds may be related to fruit formation. In *Phytelephas*, the fruit is in clusters, consisting of multiple single fruits, which may contain multiple seeds; in the tribe Borasseae, the endocarp of the fruit is composed of three separate hard pyrenes, and the seeds may be numbered one to three (Dransfield et al., 2008). Independent evolutionary events may exist in Arecoideae, with species in the tribe Coceae evolving one to three seeds in varying numbers. The size of seeds may be influenced by the size and structure of the plant and is a major determinant of seed dispersal, seedling growth, and plant evolution (Moles, 2018).

The number of stamens in Arecaceae shows a wide diversity, with numbers ranging from dozens to hundreds **(** **Table 5** **)**. In the study, the number of stamens in ancestral species of the Arecaceae may have been six, while later evolving to more numerous stamens. The number of stamens in Coryphoideae and Arecoideae species are more diverse relative to other subfamily species. *Phytelephas*, in Ceroxyloideae, possesses both numerous stamens and dioecious species. The number of stamens in this genus varies from 36 to more than 900 stamens, which is the highest number known in the family (Uhl and Moore, 1977; Dransfield et al., 2008). The number of stamens may be related to the expansion and morphological changes in the apical part of the flower prior to germination, and stamen centrifugal development appears to be a method of apical expansion to accommodate the increased number of stamens (Uhl and Moore, 1977). In Calamoideae, species of *Eugeissona* have a large number of stamens, which can upwards of 70. Stauffer et al. (2016) showed that *Eugeissona* exhibits a range of reproductive characteristics that are generally unique among the early differentiating subfamilies, and that the pistil of this genus have an unusual structure in terms of carpel fusion and differentiation for the Aeacaceae. *Nypa* in Nypoideae and *Areca* in Arecoideae showed degeneration in the number of stamens, which was reduced to three. The number of stamens in *Nypa* was influenced by its morphology and the vascular system, with filaments innately fused and anthers fused adaxially to the connectives (Uhl, 1972). In any case, the decrease or increase in the number of stamens represents a state of derivation.

## Conclusions

In this study, we assembled the complete chloroplast genomes of 24 Arecaceae species, providing a genomic resource for future research. To better understand Arecaceae, we analyzed and compared the chloroplast genome structural features of Arecaceae, inferred phylogenetic relationships, estimated the divergence time of Arecaceae, and reconstructed the analysis of ancestral traits. Based on the phylogenetic relationships of the whole plastome and multiple datasets analyzed by ML/BI, all five subfamilies were supported as monophyletic, the relationship between subfamilies was strongly supported, and the relationship between some tribes/genera was also well support. In addition, the estimation of the divergence time of Arecaceae shows that the crown age of Arecaceae was 96.60 [84.90-107.60] Ma in the Late Cretaceous, and the stem age was 102.40 [93.44–111.17] Ma. Through the analysis of the ancestral traits of Arecaceae, we can infer that the ancestral form was monoecious, with a single seed, six stamens, and a smooth pericarp. The chloroplast genome resources obtained in this study will be helpful for future studies on species identification and evolution, genetic diversity, and phylogeny of Arecaceae. However, the phylogenetic analyses of this study still had had some limitations. Future studies need to expand the acquisition of samples, and increase the data availability of whole chloroplast genomes, and use nuclear data to support the inferred relationships on a large scale. To this end, we can more comprehensively analyze and discuss the phylogeny and evolution of Arecaceae.

## Data availability statement

The datasets presented in this study can be found in online repositories. The names of the repository/repositories and accession number(s) can be found in the article/**Supplementary Material**.

## Author contributions

D-JC performed all molecular experiments, analyzed the data, and wrote the manuscript; H-XW, Q-HS, QW assisted in analyzing the data; JL helped run the data analysis and revise the manuscript; H-FW conceived and directed the study and revised the manuscript. All authors contributed to the article and approved the submitted version.

## Funding

This study was funded by Hainan Province Science and Technology Special Fund (ZDYF2022XDNY190), the Project of Sanya Yazhou Bay Science and Technology City (Grant number: SCKJ-JYRC-2022-83, HNF202222), and Hainan Provincial Natural Science Foundation of China (421RC486).

## Acknowledgments

We are sincerely thank H-XW, Q-HS, L-YG, X-FZ, X-RK, J-HW and X-LC for their kind help and experimental samples in this study, as well as for their help in molecular experiments and data analysis; thanks to Drs. L-XG and QC for their help in species identification; and thanks to H-FW and JL for their unique comments and suggestions, and for improving the manuscript; meanwhile thanks to the reviewers for their thoughtful comments and suggestions to us.

## Conflict of interest

Author QW is employed by Hainan Shengda Modern Agriculture Development Co., Ltd., Qionghai, China.

The remaining authors declare that the research was conducted in the absence of any commercial or financial relationships that could be construed as a potential conflict of interest.

## Publisher’s note

All claims expressed in this article are solely those of the authors and do not necessarily represent those of their affiliated organizations, or those of the publisher, the editors and the reviewers. Any product that may be evaluated in this article, or claim that may be made by its manufacturer, is not guaranteed or endorsed by the publisher.

## Supplementary material

Supplementary Figure S1 Multiple genome alignments visualization of chloroplast genome sequence in Arecaceae.

Supplementary Figures S2–7 The S2–S7 are the phylogenetic relationships obtained by ML and BI analysis. Based on six datasets such as LSC, SSC, IRB, NO-IRa, CDS, and Non-CDS. “*” means 100%/1.0 support value, “-” means bootstrap value/Bayesian posterior probability less than 60/0.6. Different colors represent different clades of subfamilies.

## Abbreviations

BI, Bayesian Inference; CTAB, Cetyltrimethylammonium bromide; IR, Inverted repeat; LSC, Large single copy; GTR, General time reversible; ML, Maximum Likelihood; PI, Phylogenetic informativeness; rRNA, Ribosomal RNA; SSC, Small single copy; tRNA, Transfer RNA.
