# Supplementary figures and images for: Plastome structure, phylogenomic analyses and molecular dating of Arecaceae
Source: Front Plant Sci. 2022 Sep 27;13:960588. doi: 10.3389/fpls.2022.960588 (PMC9552784; doi:10.3389/fpls.2022.960588)

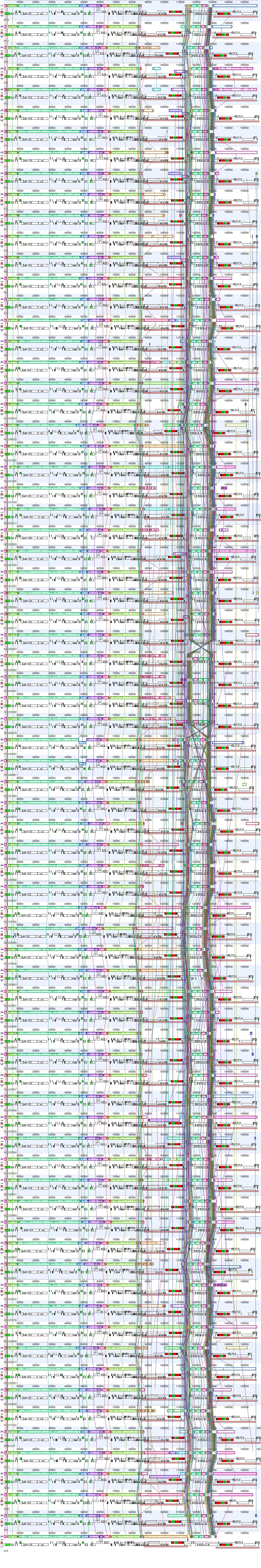

Supplement: Supplementary Figure S1 — Multiple genome alignments visualization of chloroplast genome sequence in Arecaceae. [file Image_1.jpeg]

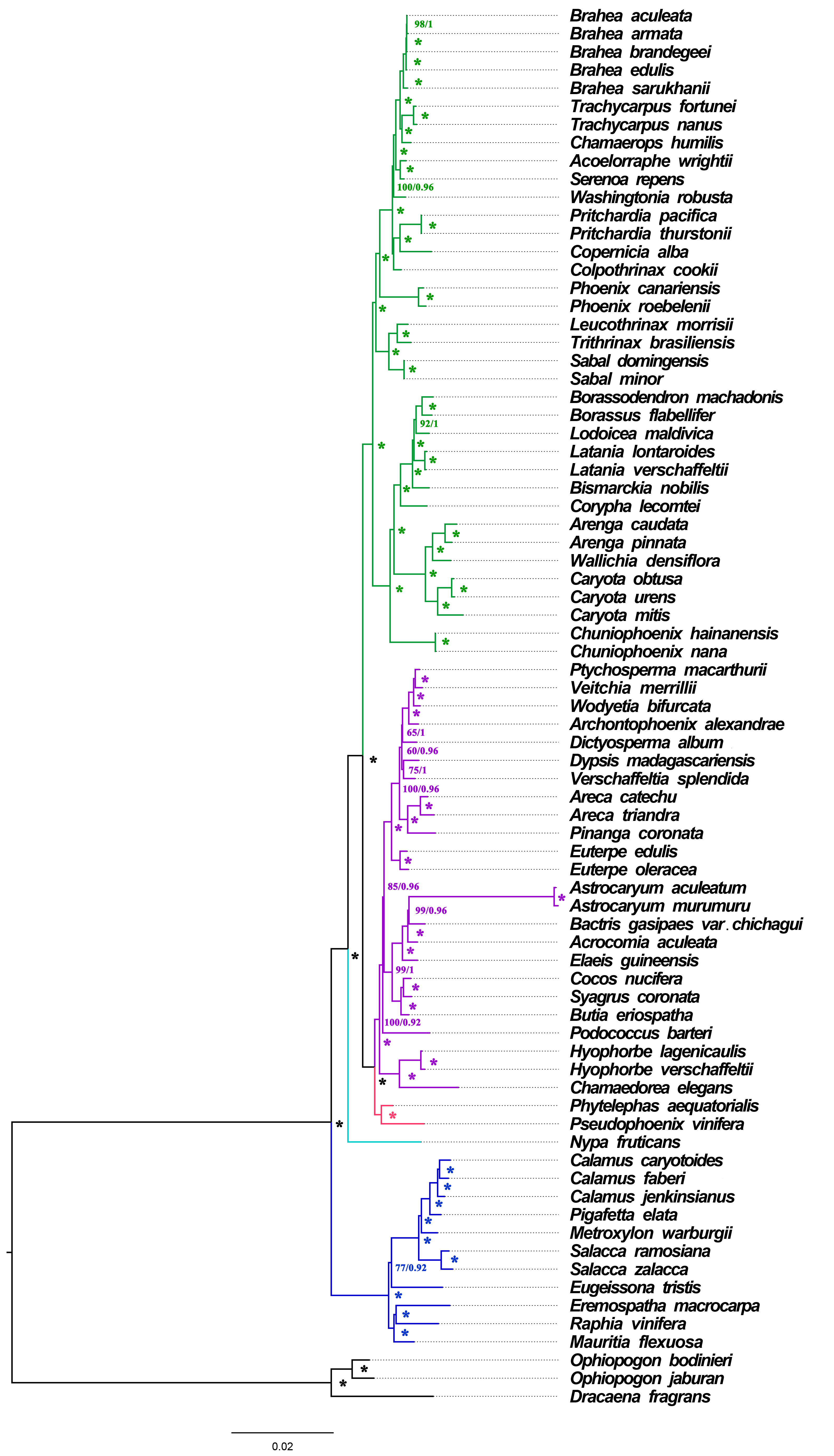

Supplement: Supplementary Figures S2–7 — The S2–S7 are the phylogenetic relationships obtained by ML and BI analysis. Based on six datasets such as LSC, SSC, IRB, NO-IRa, CDS, and Non-CDS. “*” means 100%/1.0 support value, “-” means bootstrap value/Bayesian posterior probability less than 60/0.6. Different colors represent different clades of subfamilies. [file Image_2.jpg]

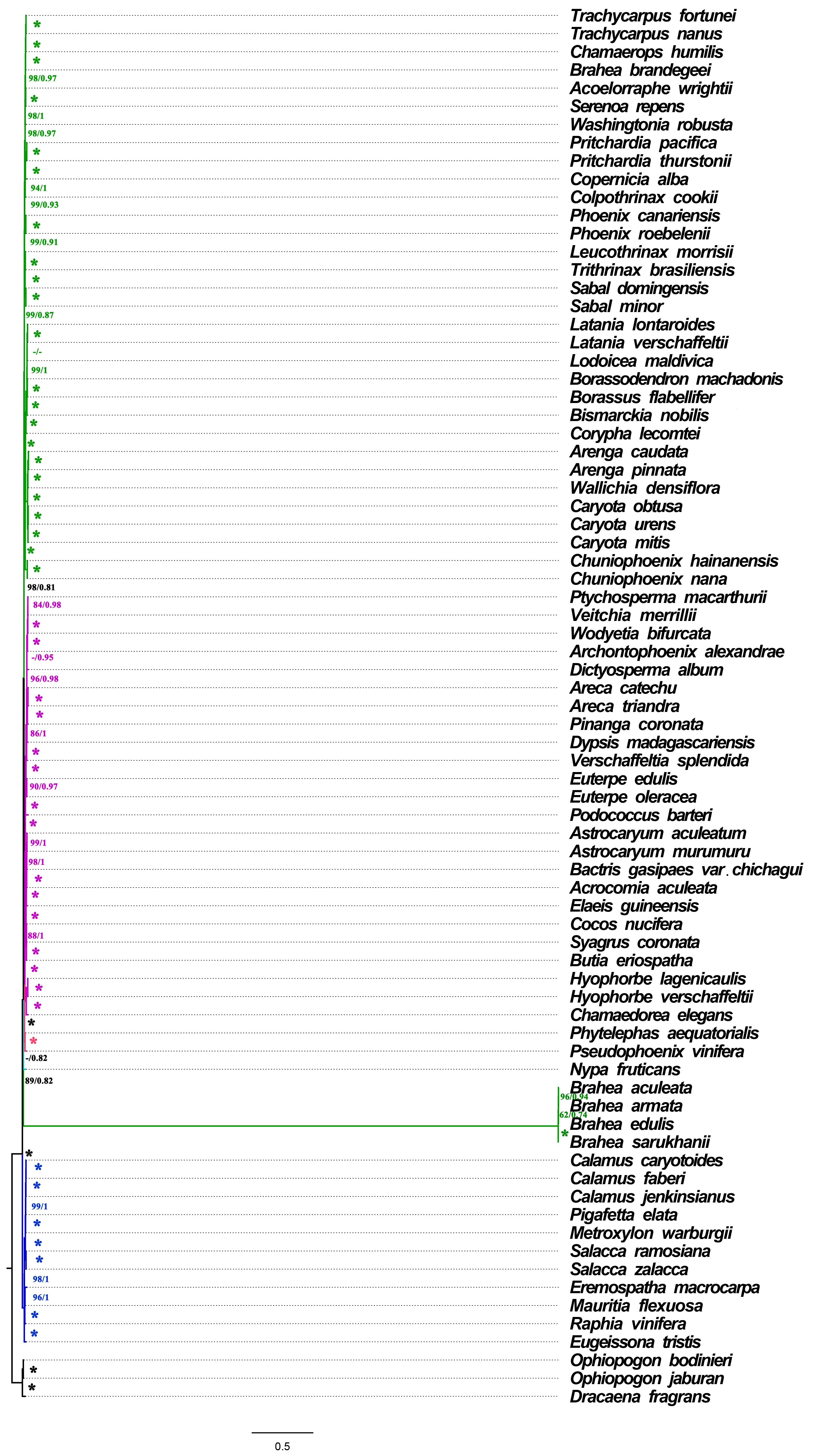

Supplement: Supplementary file 3 [file Image_3.jpg]

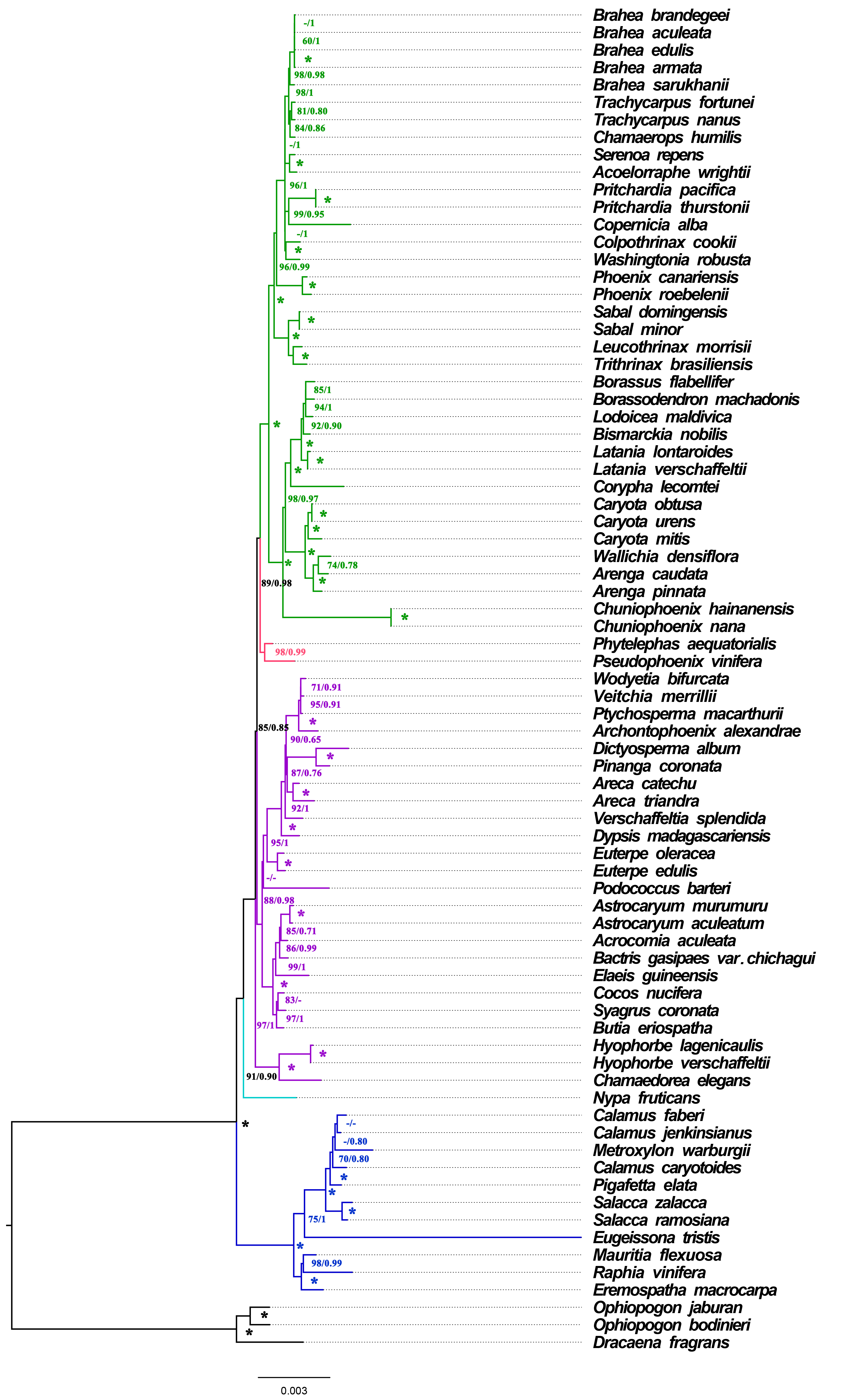

Supplement: Supplementary file 4 [file Image_4.jpg]

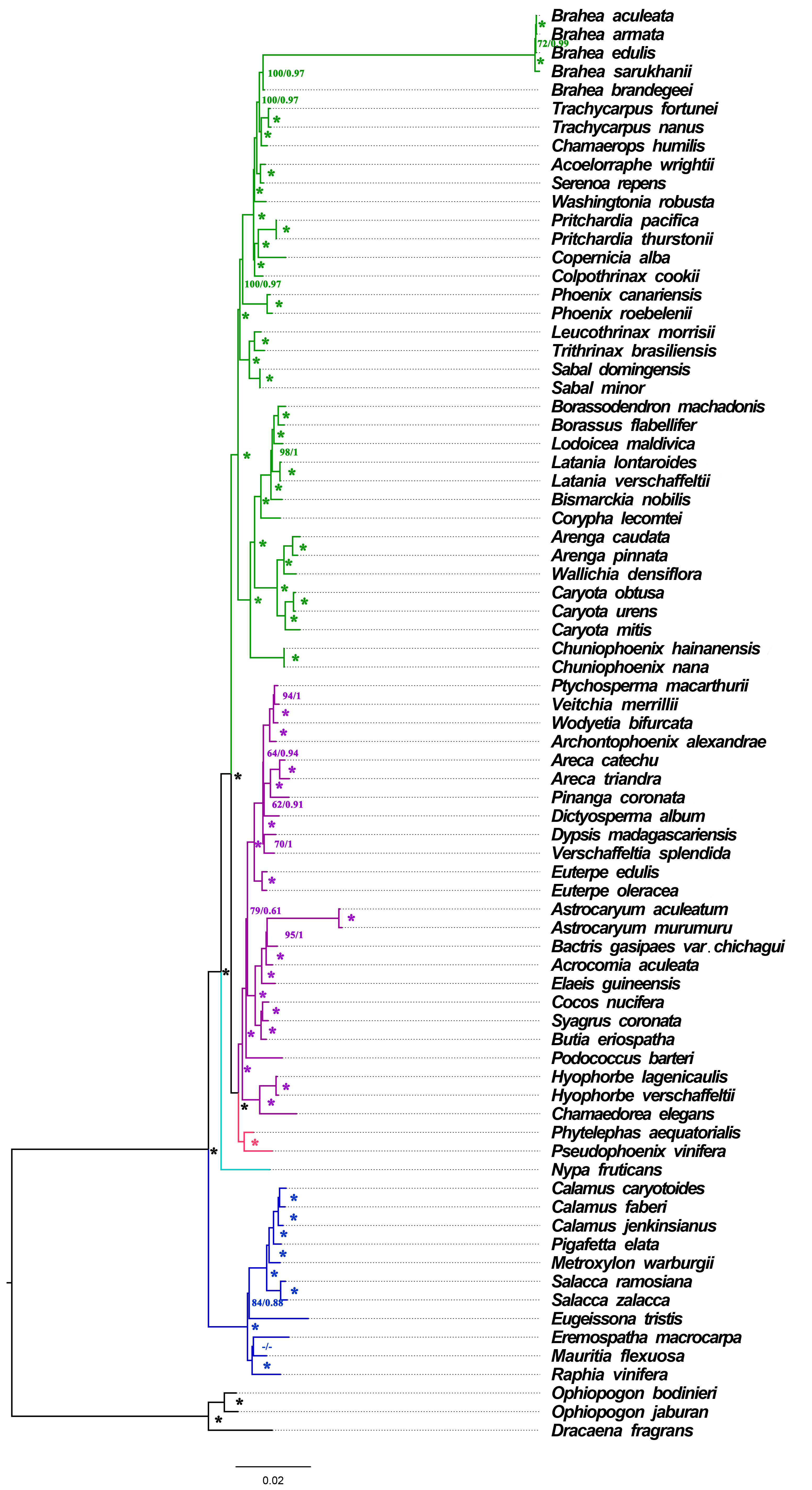

Supplement: Supplementary file 5 [file Image_5.jpg]

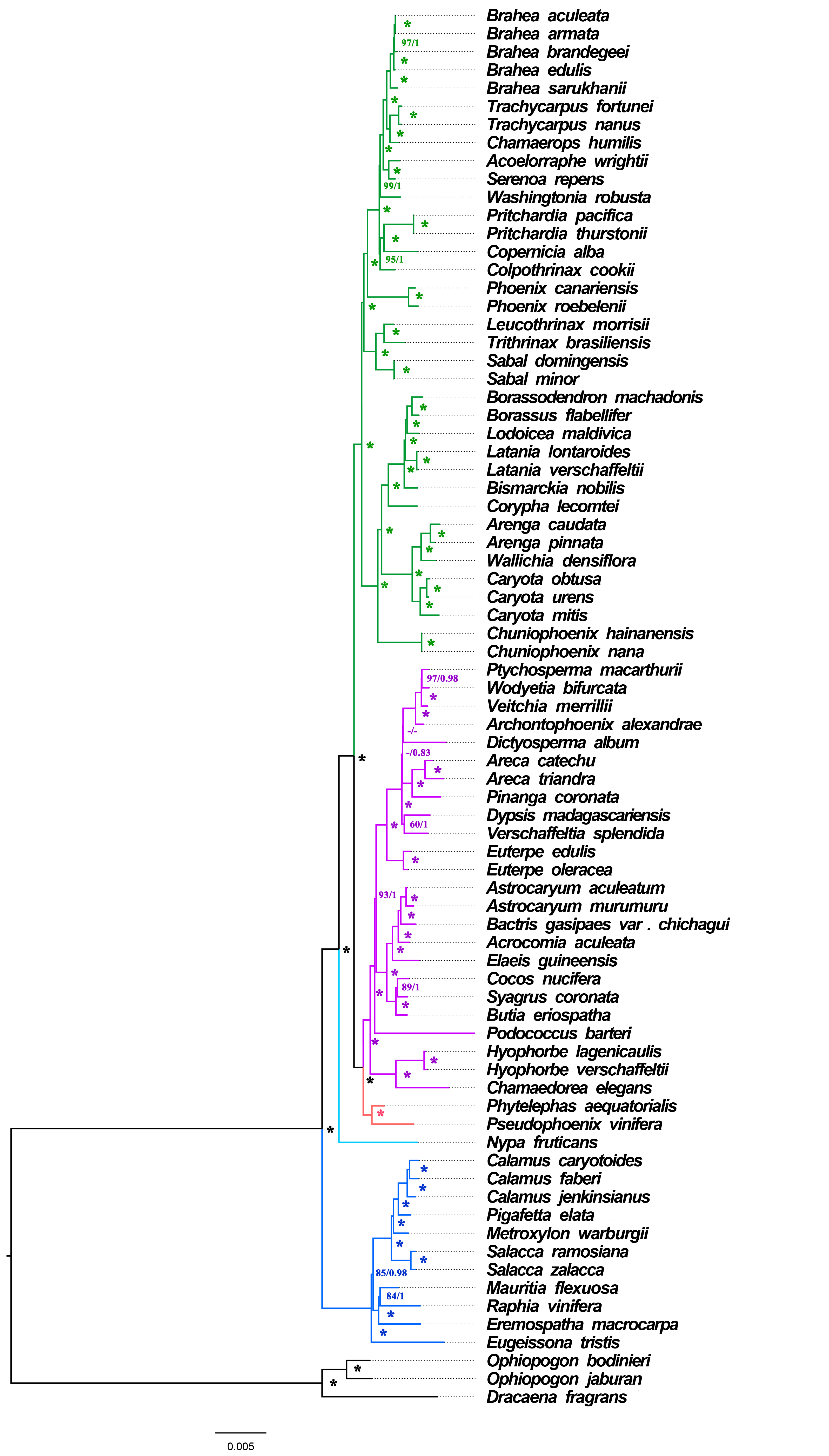

Supplement: Supplementary file 6 [file Image_6.jpg]

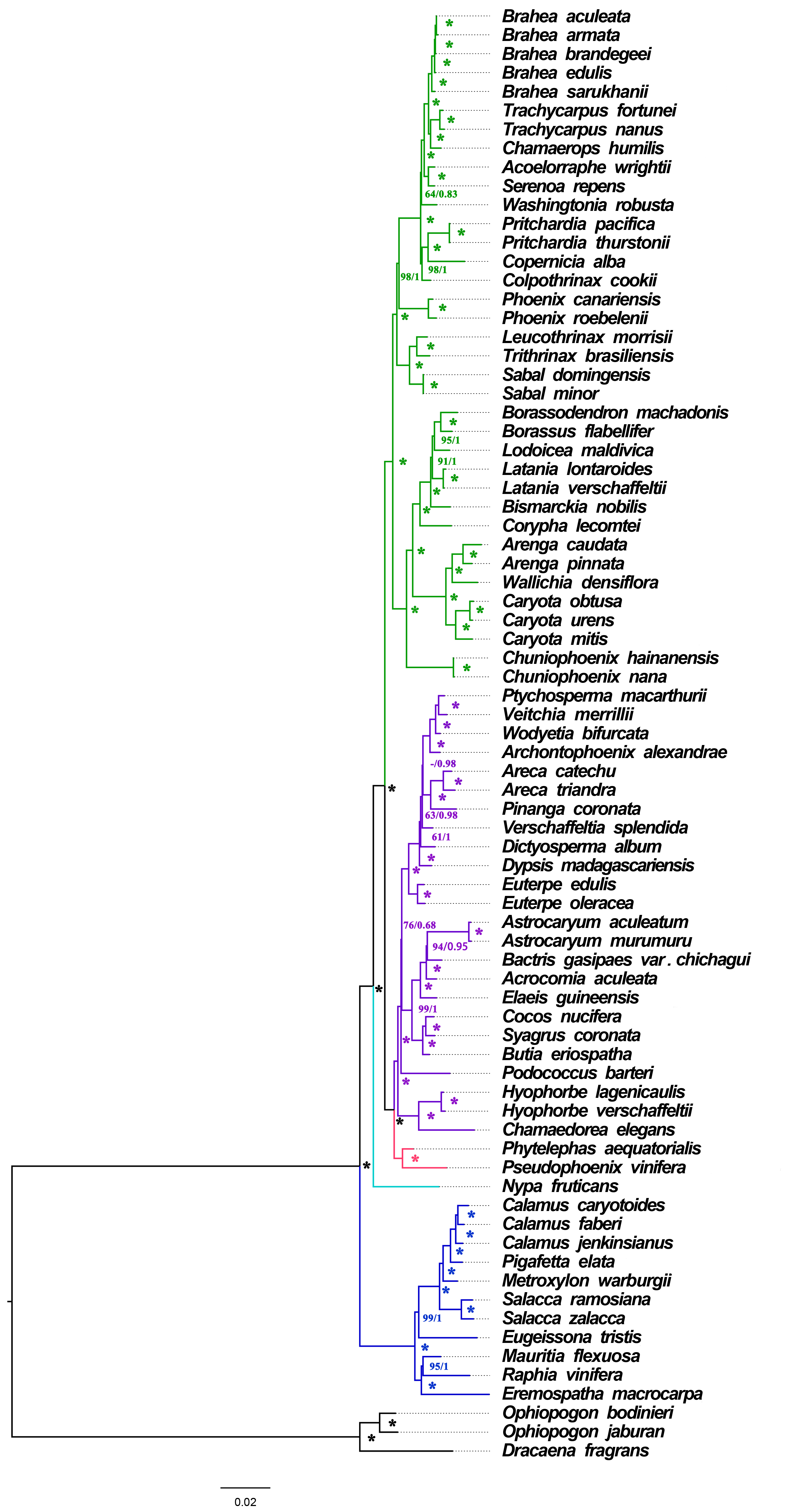

Supplement: Supplementary file 7 [file Image_7.jpg]
